# Eppikajutsuto for Treatment of Lymphatic Malformations in Children: A Nonrandomized Clinical Trial

**DOI:** 10.1001/jamanetworkopen.2025.40897

**Published:** 2025-11-03

**Authors:** Keiko Ogawa-Ochiai, Seisho Sakai, Isamu Saeki, Yuko Tazuke, Shuichiro Uehara, Akihiro Fujino, Taiki Nozaki, Hongyang Li, Motonari Nomura, Reina Hoshi, Naoki Shimojima, Junko Ochi, Shimpei Akiyama, Sho Kurihara, Kei Oyama, Hideaki Sato, Akihiro Kawahara, Kenichi Yoshimura, Yasushi Orihashi, Keigo Osuga, Hideki Ishikawa

**Affiliations:** 1 Kampo Clinical Center, Hiroshima University Hospital, Hiroshima, Japan; 2 Department of Pediatric Surgery, Kanazawa University Hospital, Kanazawa City, Japan; 3 Department of Pediatric Surgery, Hiroshima University Hospital, Hiroshima City, Hiroshima, Japan; 4 Department of Pediatric Surgery, Osaka University Graduate School of Medicine, Osaka, Japan; 5 Pediatric Surgery, Hyogo Medical University, Hyogo, Japan; 6 Department of Pediatric Surgery, Nihon University School of Medicine, Tokyo, Japan; 7 Department of Pediatric Surgery, Keio University School of Medicine, Tokyo, Japan; 8 Department of Radiology, Keio University School of Medicine, Tokyo, Japan; 9 Pediatric Surgery, University of Osaka, Osaka, Japan; 10 Division of Surgery, Department of Surgical Specialties, National Center for Child Health and Development, Tokyo, Japan; 11 Department of Radiology, University of Iowa Hospitals and Clinics, Iowa City; 12 Department of Radiology, Kyoto Prefectural University of Medicine, Kyoto, Japan; 13 Department of Pediatric Surgery, St Marianna University School of Medicine, Kawasaki, Japan; 14 Department of Pediatric Surgery, Showa Medical University Hospital, Tokyo, Japan; 15 Center for Integrated Medical Research, Hiroshima University Hospital, Hiroshima, Japan; 16 Clinical Research Center in Hiroshima, Hiroshima University Hospital, Hiroshima, Japan; 17 Department of Diagnostic Radiology, Osaka Medical and Pharmaceutical University, Osaka, Japan; 18 Department of Molecular Targeting Prevention, Graduate School of Medical Science, Kyoto Prefectural University of Medicine, Osaka, Japan

## Abstract

**Importance:**

Lymphatic malformations (LMs) are congenital vascular anomalies with limited treatment options. Eppikajutsuto, a traditional Japanese (Kampo) herbal medicine, is commonly used to treat inflammatory conditions and has been reported to reduce lesion volume in patients with LMs; however, data on its effectiveness in pediatric patients are needed.

**Objective:**

To evaluate the association of eppikajutsuto with reduced LM lesion volume in pediatric patients with LMs.

**Design, Setting, and Participants:**

This open-label nonrandomized clinical trial was conducted from April 14, 2021, to September 30, 2024, at multiple centers in Japan. Eligible participants were 18 years or younger with a confirmed diagnosis of LM by imaging. Data were analyzed from October 14, 2024, to March 4, 2025.

**Intervention:**

Participants received eppikajutsuto granules (0.6 g/kg/d to ≤7.5 g/d) orally for 6 months.

**Main Outcomes and Measures:**

The primary outcome was the proportion of patients achieving a 20% or greater reduction in lesion volume, measured with magnetic resonance imaging volumetry at baseline and 6 months. Secondary outcomes included a 50% or greater reduction in lesion volume, median shrinkage rate, improvement in quality of life, and safety.

**Results:**

A total of 19 patients (10 male [52.6%]; mean [SD] age, 24.1 [29.4] months) were enrolled. A total of 10 patients (52.6%; 90% CI, 32.0%-73.0%) achieved a 20% or greater reduction in lesion volume at 6 months. The median shrinkage rate was 22.9% (IQR, −142.3% to 96.0%). No serious adverse events occurred, and medication adherence of 70% or greater was observed in 17 of 19 patients (89.5%).

**Conclusions and Relevance:**

In this nonrandomized clinical trial of children with LMs, eppikajutsuto was associated with a reduction in lesion volume and was well tolerated. Further studies, including randomized clinical trials, are warranted to confirm these findings.

**Trial Registration:**

Japan Registry of Clinical Trials Identifier: jRCTs041210007

## Key Points

**Question:**

Is eppikajutsuto, a traditional Japanese (Kampo) herbal medicine, associated with reduced lesion volume among children with lymphatic malformations (LMs)?

**Findings:**

In this nonrandomized clinical trial of 19 children with LMs, 10 (53%) achieved a 20% or greater reduction in lesion volume at 6 months. No serious adverse events were reported.

**Meaning:**

These findings suggest that eppikajutsuto was associated with a reduction in lesion volume and was well tolerated in children with LMs.

## Teaser

This nonrandomized clinical trial evaluates the association of eppikajutsuto, a traditional Japanese (Kampo) herbal medicine, with lesion size in pediatric patients with lymphatic malformations.

## Introduction

Lymphatic malformations (LMs) are congenital vascular anomalies defined by the International Society for the Study of Vascular Anomalies.^1^ They result from abnormal development of the lymphatic system, leading to the formation of fluid-filled cystic lesions. LMs frequently occur in the head and neck region and are associated with substantial morbidity, owing to their tendency to increase over time. This enlargement can lead to functional impairment, cosmetic deformity, and complications such as infection and airway obstruction. In particular, LMs in the cervical or mediastinal region may cause bronchial compression in neonates and children, whereas lesions in the extremities may restrict mobility or cause pain, thereby affecting daily activities.

Although the exact incidence of LMs remains uncertain, their estimated prevalence is approximately 1 in 4000 live births.^2^ Standard therapies covered under Japanese National Health Insurance include sclerotherapy and surgical resection. Sclerotherapy is particularly effective for cystic LMs; however, it can cause temporary swelling and compression of adjacent vital structures, including the airway. Surgical resection is technically demanding, particularly in anatomically complex regions. Therefore, less invasive treatment options are required. Sirolimus, a mechanistic target of rapamycin (mTOR) inhibitor, is approved for the treatment of LMs in the US and other countries.^3^ In Japan, sirolimus was approved in 2023 but only for intractable cases, following multiple reports of efficacy.^4^ However, sirolimus use is limited by the risk of serious adverse events, including thrombocytopenia, lymphopenia, hyperlipidemia, infection, and cardiac complications. Moreover, the cost of sirolimus is considerable (approximately ¥1308.80 [US $8.84] per milligram), with daily dosages between 1 and 4 mg.^5^

Kampo medicine, the traditional Japanese system of herbal medicine, is derived from ancient Chinese medicine but has evolved under Japanese cultural and clinical influences. Kampo formulas are included in the Japanese national formulary and widely used in clinical practice. Eppikajutsuto has been reported to reduce lesion volume in patients with LMs, including previous reports of the first known case of mediastinal LM that responded to eppikajutsuto^6^ and a neonatal cervical LM showing marked regression after dosage adjustment.^7^ Other case reports have also suggested the therapeutic potential of eppikajutsuto.^8,9^ In a prior retrospective study, partial responses to eppikajutsuto were documented, although radiologic evaluation was not standardized.^10^

Eppikajutsuto was approved by the Ministry of Health, Labour and Welfare of Japan in 1986 and is regulated by the Japan Society for Oriental Medicine.^11^ It is one of the most commonly used Kampo prescriptions for inflammatory conditions such as allergies and infections. Historically, eppikajutsuto is derived from an ancient Chinese medical text^12^ and has been used to reduce edema and improve water retention. In pediatric practice, eppikajutsuto is used for nocturia, with a long-standing safety record; no serious adverse events have been reported.

Eppikajutsuto is also cost-effective, priced at approximately ¥16.10 (US $0.11) per gram, with pediatric dosing between 0.3 and 0.7 g/kg/d (≤7.5 g/d), totaling approximately ¥120.75 (US $0.82) daily.^2^ The ephedrine content of eppikajutsuto is very low (approximately 0.7% combined pseudoephedrine and ephedrine), and adverse effects are rare under medical supervision. Despite these promising observations, no prospective studies have evaluated the efficacy, dose response, or safety profile of eppikajutsuto in the treatment of LMs.

We conducted a prospective trial to evaluate the association of eppikajutsuto with reduction in lesion size in pediatric patients with LMs using magnetic resonance imaging (MRI)–based volumetric analysis as a primary end point. We hypothesized that oral administration of eppikajutsuto (0.6 g/kg/d to ≤7.5 g/d) for 6 months would result in a 20% or greater reduction in MRI-based lesion volume in children with LMs, with an acceptable safety profile.

## Methods

### Trial Design and Oversight

The LMEP (A Clinical Study Evaluating the Effect of Eppikajutsuto on Lymphatic Malformations) open-label nonrandomized clinical trial was conducted at multiple centers in Japan between April 14, 2021, and September 30, 2024. The trial was approved by the Kanazawa University Institutional Review Board and was conducted in accordance with the Declaration of Helsinki.^13^ Written informed consent was obtained from all participants or their guardians. Data collection was managed by medical research support staff, and analyses were performed at Hiroshima University. The trial protocol and statistical analysis plan are provided in Supplement 1. The study followed the Transparent Reporting of Evaluations With Nonrandomized Designs (TREND) reporting guideline.

### Patients

The inclusion criteria were as follows: (1) a confirmed diagnosis of an LM with characteristic cystic or cavernous lesions identified by imaging (ultrasonography, computed tomography, or MRI) and (2) body weight of up to 25 kg. Eligible patients weighed up to 25 kg to ensure dosing within the pediatric safety range (0.6 g/kg/d to ≤7.5 g/d). This weight also reflects the typical patient population with LMs, most of whom are diagnosed prenatally or in early infancy when body weight is below this threshold. Given the favorable safety profile of eppikajutsuto, it may be considered earlier than other treatment modalities, making this population a realistic target for clinical application.

The exclusion criteria were as follows: (1) history of allergic reactions or intolerance to eppikajutsuto; (2) current or prior treatment with mTOR inhibitors; (3) prior sclerotherapy or surgery for LMs; (4) presence of other serious medical conditions unrelated to LMs; (5) participation in another clinical trial within 3 months prior to enrollment; (6) inability to obtain evaluable MRI scans; and (7) any condition, as judged by the principal investigator or subinvestigator, that would make the patient unsuitable for participation.

### Interventions

Eligible patients were assigned to receive eppikajutsuto. Eppikajutsuto (TJ-28; Tsumura & Co) at a dosage of 0.6 g/kg/d (to ≤7.5 g/d) was administered orally to patients for a duration of 6 months following enrollment. The eppikajutsuto granule formulation is manufactured under Good Manufacturing Practice standards, and its quality, safety, and composition are regulated by Japan’s Ministry of Health, Labour and Welfare and the National Institutes of Biomedical Innovation, Health and Nutrition, ensuring lot-to-lot consistency of active constituents.^11^ A 7.5-g portion of eppikajutsuto extract granules contains 5.0 g of dried extract of the crude agents listed in the eTable in Supplement 2.

### Radiologic Evaluation

The effects of eppikajutsuto were assessed with the percentage of reduction in lesion volume, which was defined as a change in total lesion volume by MRI volumetry 6 months after treatment initiation. Fat-saturated T2-weighted MRI scans were obtained and analyzed using an OsiriX DICOM viewer, version 9.0 (Pixmeo). Volumetry was performed semiautomatically with a region of interest (ROI) tool. If the lesion shape was complex, manual segmentation using closed polygon ROIs was used. Volumes were calculated by multiplying the ROI areas by the slice thickness.

The sequences required were T1 weighted, T2 weighted, fat-suppressed T2 (T2FS), and 3-dimensional T2FS (3D-T2FS). Therefore, axial views were mandatory. T2FS images were acquired without any gaps. The slice thickness was typically 3 to 5 mm, extendable to 6 to 8 mm for larger lesions. The final evaluations were based on volumetric comparisons using 3D-T2FS. If unavailable, gapless T2FS was substituted.

Two independent radiology specialists (J.O. and S.A.), serving as central evaluators, reviewed all patient results. Lesion boundaries were confirmed in multiple planes (eg, axial and coronal), and ROIs were drawn accordingly, with volume measurements performed primarily on axial images. Each evaluator independently measured the lesion volume, and the mean of the 2 measurements was considered as the final value. All of the measurements were rounded to their nearest tenth.

All MRI scans were evaluated by 2 independent central radiologists (J.O. and S.A.) blinded to clinical data, including treatment duration, patient characteristics, and time points. Each radiologist independently performed volumetric analysis using OsiriX software, and the final measurement was calculated as the average of both. Interobserver agreement was assessed using the intraclass correlation coefficient (ICC). Interobserver reproducibility was assessed using the ICC to ensure measurement reliability. This centralized, blinded evaluation approach aimed to ensure objectivity and minimize site-specific variability in imaging assessments.

### Trial End Points

The primary end point was response rate, defined as a 20% or greater reduction in lesion volume of LMs at 6-month follow-up based on MRI volumetry, or main lesion shrinkage in diffuse cases. This criterion was selected based on prior clinical observations, which suggested measurable changes within 6 months of treatment.

Secondary end points were evaluated at 6 months and included the following: (1) 50% or greater reduction in lesion volume; (2) mean shrinkage rate; (3) safety; (4) adherence (defined as ≥70% of prescribed doses taken); and (5) a 10% or greater improvement in quality of life (QOL). QOL was assessed in patients 5 years or older using the validated Japanese version of the EQ-5D-Y caregiver-proxy questionnaire.

### Statistical Analysis

In a previous retrospective observational study,^10^ the response rate to eppikajutsuto therapy for LMs (defined as a ≥20% reduction in volume) was 83% in lesion volume on MRI in 8 pediatric patients with LMs, with no serious adverse events reported. These preliminary findings suggested the potential effectiveness of eppikajutsuto and a favorable safety profile, warranting prospective evaluation. Acknowledging the tendency for overestimation in retrospective designs, we conservatively expected a response rate of 50% or greater in this prospective trial. In a previous study,^10^ untreated case patients showed progressive lesion enlargement with no spontaneous regression observed. Given the exploratory nature of the current prospective trial, the sample size was calculated to assess the precision of the estimated response rate. If 9 of the 18 patients responded (50%), the lower limit of the 2-sided 90% CI would be 29.1% with an upper limit of 70.8%, assuming a 1-sided significance level of 5%. This was considered sufficient to support future hypothesis-driven trials. Allowing for possible exclusions, the final sample size was set at 20.

Primary analysis was conducted using the full analysis set, excluding patients who were ineligible, met the exclusion criteria, did not receive protocol treatment, or lacked data after enrollment. For the primary end point, the response rate was calculated as the proportion of patients achieving a 20% or greater reduction in LM lesion volume at 6-month follow-up, assessed via MRI volumetry. Interval estimates were obtained using an exact method based on a binomial distribution with a 1-sided significance level of 5% and corresponding 2-sided 90% CI.

Subgroup analyses were performed according to sex, lesion site, medication adherence (≥70% vs <70%), and age (<1 year vs ≥1 year at enrollment). Adherence-based subgroup analysis was exploratory and descriptive in nature. No adjustments for time-varying confounding were applied.

The study data were analyzed from [Month] [Day], [Year], to [Month] [Day], [Year]. Statistical analyses were performed using JMP, version 17 (JMP Statistical Discovery LLC), and SAS, version 9.4 (SAS Institute Japan Inc).

## Results

### Patient Characteristics

**Figure 1.**
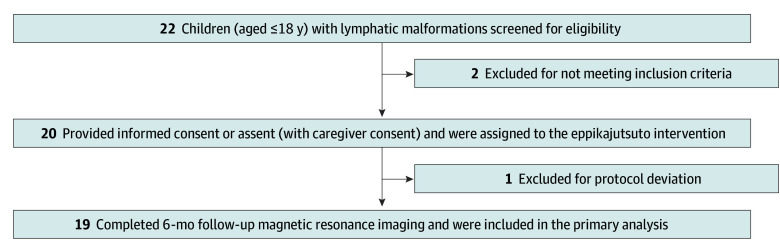
Flow Diagram of Patient Enrollment, Screening, and Analysis The final analysis set comprised patients who received at least 1 dose of eppikajutsuto and had evaluable magnetic resonance imaging volumetric data at the 6-month follow-up. Long-term outcomes at 12 months were evaluated based on imaging and clinical follow-ups in a subset of patients.

Of the 20 patients recruited, 1 was excluded for protocol deviation due to a dosage error (Figure 1). Therefore, 19 children with LMs (9 female [47.4%] and 10 male [52.6%]; mean [SD] age, 24.1 [29.4] months) were prospectively enrolled in this trial. Patient characteristics and treatment are summarized in Table 1. Thirteen patients (68.4%) had a body weight of less than 12.5 kg. Six patients (31.6%) had lesions in the head and neck areas. All patients had at least 1 target lesion that was measurable using MRI and were examined at the 6-month follow-up. Although the primary analysis was conducted at 6 months, exploratory follow-up data at 12 months were collected for patients who continued eppikajutsuto treatment and had available imaging.

**Table 1.** Patient Characteristics^a^

Characteristic	Values (N = 19)
Sex	
Female	9 (47.4)
Male	10 (52.6)
Age, mean (SD), mo	24.1 (29.4)
Height, mean (SD), cm	76.4 (24.9)
Weight, mean (SD), kg	
<12.5 (n = 13 [68.4])	6.5 (2.4)
≥12.5 (n = 6 [31.6])	17.6 (4.1)
Total	10.0 (6.0)
Baseline lesion volume, mean (SD) [range], cm^3^	45.2 (38.7) [8.5-160.3]
Existing medical history^b^	3 (15.8)
Complications^c^	2 (10.5)
Type of lymphatic malformation	
Macrocystic	3 (15.8)
Microcystic	1 (5.3)
Mixed	15 (79.0)
Lesion site	
Head and neck	6 (31.6)
Body trunk	9 (47.4)
Extremities	4 (21.1)

^a^ Unless indicated otherwise, values are presented as No. (%) of patients.

^b^ Included febrile convulsions, bronchial asthma, and right inguinal hernia.

^c^ Included median cervical cyst, as well as left gluteal hemangioma and right preauricular fistula.

### Primary End Point

**Figure 2.**
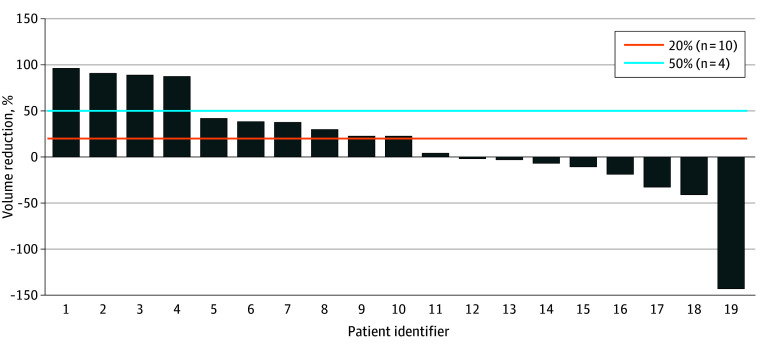
Volumetric Response to Eppikajutsuto in All Patients With Lymphatic Malformations

Figure 2 illustrates volumetric changes in patients who underwent radiologic examination. Of the 19 patients, 10 (52.6%; 90% CI, 32.0%-73.0%) had a 20% or greater reduction in lesion volume.

**Figure 3.**
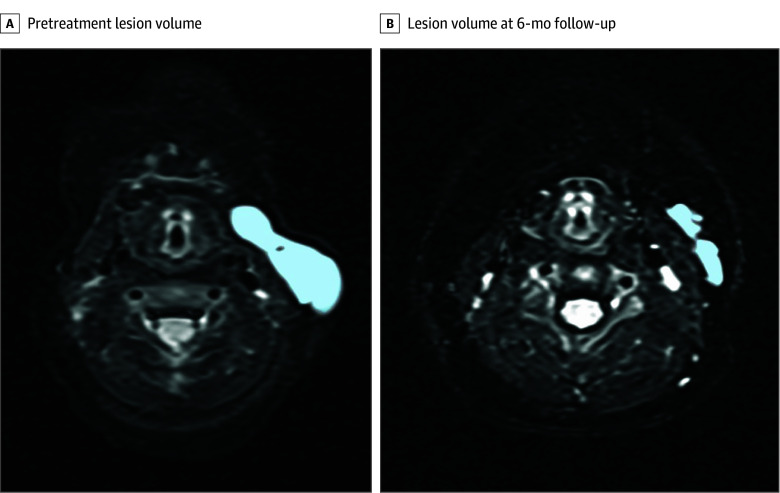
Magnetic Resonance Imaging (MRI)–Based Volumetric Analysis of a Representative Patient MRI (T2-weighted) scan of a 1-month-old infant with a lymphatic malformation on the anterior surface of the sternocleidomastoid muscle. A, Pretreatment MRI scan showing a cystic lesion with a volume of 4.71 cm^3^. B, MRI performed at 6-month follow-up after initiating eppikajutsuto revealed a marked reduction, with a final lesion volume of 0.19 cm^3^. Volumetric measurements were obtained using an OsiriX DICOM viewer.

MRI scans from a representative case patient (a 1-month-old child with a cervical LM) with a marked volumetric response to eppikajutsuto are shown in Figure 3. The patient demonstrated a reduction in lesion volume from 4.71 cm^3^ at baseline to 0.19 cm^3^ at the 6-month follow-up, as measured using MRI-based volumetry. The ICC for interobserver agreement in MRI volumetry was 0.96 (95% CI, 0.93-0.98), demonstrating excellent reproducibility.

### Secondary End Points

A 50% or greater reduction in lesion volume was observed in 4 of 19 patients (21.1%; 90% CI, 7.5%-41.9%). The median percentage change in lesion volume was 22.9% (IQR, −142.3% to 96.0%). QOL improvement was assessed in only 3 patients older than 5 years; 2 patients (66.7%) achieved a 10% or greater improvement in QOL scores. Medication adherence of 70% or greater was observed in 17 of 19 patients (89.5%).

Subgroup analyses of the primary response rate (≥20% volume reduction) are presented in Table 2. Males had a higher response rate (n = 7 of 10 [70.0%]; 90% CI, 39.3%-91.3%) than females (n = 3 of 9 [33.3%]; 90% CI, 9.8%-65.5%). Response rates were also higher in patients with head and neck lesions (n = 5 of 6 [83.3%]; 90% CI, 41.8%-99.1%) than in patients with lesions in other locations (n = 5 of 13 [38.5%]; 90% CI, 16.6%-64.5%). Among the 17 patients with 70% or greater medication adherence, 10 (58.8%; 90% CI, 36.4%-78.8%) experienced a treatment response. Of the 9 patients aged 1 year or older at enrollment, 6 (66.7%; 90% CI, 34.5%-90.2%) experienced a response.

**Table 2.** Subgroup Analysis

Subgroup	Response rate (90% CI), %
Sex	
Female	33.3 (9.8-65.5)
Male	70.0 (39.3-91.3)
Lesion site	
Head and neck	83.3 (41.8-99.1)
Other	38.5 (16.6-64.5)
Medication adherence ≥70%	58.8 (36.4-78.8)
Age >1 y at consent	66.7 (34.5-90.2)

A total of 11 patients (57.9%) continued eppikajutsuto treatment beyond 6 months and had data available at 12-month follow-up. Of these, 1 patient who temporarily discontinued the treatment experienced lesion progression and subsequently resumed therapy. MRI was performed in only 4 patients (36.4%); the remaining 7 patients (63.6%) were assessed via ultrasonography or clinical evaluation. Among the evaluable patients, 3 (27.3%) showed further lesion reduction, 2 (18.2%) underwent sclerotherapy, and 1 (9.1%) required surgical resection.

### Safety

Adverse events occurred in 3 of the 19 patients (15.8%) during the 6-month treatment period. These events included neutropenia (grade 4), LM infection (grade 1), and hepatic dysfunction (grade 1). No fatalities were reported. Additionally, 1 patient (5.3%) developed an upper respiratory tract infection and urticaria. No adverse events were observed during the 12-month extended treatment period.

## Discussion

To our knowledge, this is the first prospective nonrandomized clinical trial to evaluate the association of eppikajutsuto with lesion size in children with LMs. Our findings suggest that eppikajutsuto was associated with a volume reduction of 20% or greater in 10 of 19 patients (52.6%) at 6-month follow-up, demonstrating its potential as a therapeutic option for LMs. Additionally, a 50% or greater volume reduction was observed in 4 of 19 patients (21.1%), further supporting its clinical relevance. Considering the limited treatment options for LMs, these results provide important insights into the therapeutic potential of eppikajutsuto. Although this study used an open-label design, the primary outcome (MRI-based volumetric change) was assessed through a blinded central radiologic review, which helped mitigate measurement bias and improve objectivity.

### Comparison With Sclerotherapy

Sclerotherapy with agents such as OK432 or doxycycline remains a standard treatment for macrocytic LMs, with reported response rates between 58% and 96%, depending on lesion characteristics and the sclerosing agent used.^14,15^ However, adverse effects are common, with temporary pain, swelling, or low-grade fever occurring in approximately 50% of patients.^14^ Cervical or mediastinal lesions have particularly high risk because of potential airway obstruction from postprocedural inflammation, occasionally requiring emergency airway management. Respiratory compromise has been reported in 5% to 7% of patients treated with OK432.^14,15^

In contrast, a partial response rate of 52.6% (n = 10 of 19 patients) was achieved with eppikajutsuto therapy in the current study. In addition, 83.3% (n = 5 of 6) of patients with head and neck lesions had a 20% or greater reduction in lesion volume. No adverse respiratory events or hospitalizations were observed. Apart from 1 grade 4 event, eppikajutsuto was administered orally on an outpatient basis without sedation or procedural risks and was generally well tolerated. Although sclerotherapy may offer faster or greater lesion shrinkage, the results of this study suggest that eppikajutsuto is a safer, noninvasive alternative, especially for patients with adjacent airway lesions or contraindications to interventional therapies.

### Comparison With mTOR Inhibitors

Sirolimus, an mTOR inhibitor, has shown clinical efficacy in reducing LM burden, with partial response rates between 55% and 65% after 12 to 52 weeks of therapy.^4^ However, adverse events, such as stomatitis, infections, hyperlipidemia, and hematologic abnormalities, have occurred in more than 80% of patients, often requiring dose adjustment or discontinuation.^4^

In our study, eppikajutsuto achieved a comparable partial response rate of 52.6% at 6-month follow-up, with a more favorable safety profile. Mild to moderate grade 1 events (LM infection, hepatic dysfunction, urticaria, and upper respiratory infection) were noted in 3 patients (15.8%), and 1 patient (5.3%) experienced a grade 4 event that was managed without discontinuation of treatment.

Importantly, 17 of 19 patients (89.5%) in this study maintained 70% or greater medication adherence, supporting the feasibility for sustained outpatient use of eppikajutsuto, even in infants. Unlike previous sirolimus studies that included patients with complex lymphatic anomalies, such as kaposiform lymphangiomatosis and Gorham-Stout disease,^4^ our study focused exclusively on LMs, which may explain the differences in tolerability and response.

No serious adverse events were observed, and eppikajutsuto treatment was generally well tolerated. However, given the limited sample size, these findings should be interpreted cautiously, and larger studies are needed to fully characterize the safety profile of eppikajutsuto.

Although the precise mechanism of action of eppikajutsuto is currently under investigation, preclinical studies suggest that it mildly modulates the mTOR pathway and exerts anti-inflammatory effects. Eppikajutsuto has been shown to downregulate mTOR protein expression without affecting extracellular signal-related kinase 1/2 signaling, and in vitro data demonstrate inhibition of cell proliferation and induction of apoptosis.^16^ Additionally, the *Ephedrae herba* alkaloids and *Gypsum fibrosum* components in eppikajutsuto may have anti-inflammatory and aquaporin-enhancing effects, respectively,^17,18^ indicating a mechanism distinct from that of sirolimus. Thus, eppikajutsuto may represent a safer and more accessible alternative for selected pediatric patients with LMs, especially those for whom immunosuppression or intensive monitoring is contraindicated.

### Factors Affecting Treatment Response

The response to eppikajutsuto varied according to patient sex and lesion location. Males had a higher partial response rate (n = 7 of 10 [70.0%]) than females (n = 3 of 9 [33.3%]), a difference that may reflect hormonal or genetic effects on lymphatic physiology. Estrogen was shown to regulate lymphangiogenesis and maintain lymphatic vessel stability via estrogen receptor–α signaling,^19^ whereas lymphatic endothelial cells demonstrated sex-specific structural and functional differences,^20^ potentially affecting disease course and treatment response.

In this study, lesion location also affected the outcomes. Patients with head and neck lesions exhibited a higher response rate (83.3%) than those with lesions in other sites (38.5%). This regional difference may be due to variations in lymphatic vessel density, lesion morphology, or proximity to systemic circulation. Head and neck lesions tend to be more superficial and cystic, potentially enhancing the delivery and effectiveness of anti-inflammatory treatments such as eppikajutsuto. Prior studies have also reported an improved treatment response for LMs in this region, likely related to its anatomical characteristics and accessibility.^21,22^

Medication adherence is another important factor. In this study, patients with 70% or greater adherence had a higher response rate (n = 10 of 17 [58.8%]; 90% CI, 36.4%-78.8%) than those with lower adherence, emphasizing the critical role of compliance in therapeutic success. Because adherence was assessed during follow-up, analyses based on this variable are prone to time-dependent bias and should be interpreted cautiously. These analyses were exploratory and were not intended to establish causality.

Because baseline lesion volumes varied widely among patients in this study, we selected the percentage of reduction as the primary end point to standardize comparisons. This approach is supported by previous LM studies using MRI volumetry in which proportional change accounted for lesion size heterogeneity.^3,4^ An exploratory analysis found no significant correlation between baseline volume and absolute change at 6 months (Spearman ρ = –0.12; *P* = .62), likely due to the small sample size.^10^ Percentage change therefore remains the most appropriate metric for assessing treatment response in this heterogeneous population.

Collectively, these findings suggest that sex, lesion site, lesion volume, and adherence should be considered when evaluating the effectiveness of eppikajutsuto and designing future treatment strategies for pediatric patients with LMs.

### Clinical Implications

This study suggests that eppikajutsuto may offer a promising alternative to mTOR inhibitors for the treatment of LMs, particularly in younger patients and those with head and neck lesions. Notably, 2 of the 3 patients (66.7%) older than 5 years in this study reported improved QOL,^23,24^ suggesting that the benefits of eppikajutsuto may extend beyond lesion reduction. Given its favorable safety profile, eppikajutsuto may be a viable option for patients who are ineligible for or unable to tolerate mTOR inhibitor treatment.

However, the continuation rate between 6 months and 12 months was modest (n = 11 of 19 [57.9%]) in this trial, which may reflect the challenges of long-term adherence to eppikajutsuto. Kampo formulations are typically granulated, which may pose palatability concerns in pediatric patients.

Targeted therapies, particularly *PIK3CA* inhibitors such as alpelisib, have emerged as promising options for treating LMs associated with the *PIK3CA*-related overgrowth spectrum.^25^ Early-phase studies reported substantial reductions in lesion volume, and randomized clinical trials are under way. However, these agents may carry adverse effects (eg, hyperglycemia, rash, or gastrointestinal symptoms), and access for pediatric patients remains limited. Given its favorable safety profile, oral administration, and cost-effectiveness, eppikajutsuto may serve as a complementary or alternative option, especially for patients who are ineligible for targeted therapies.

### Limitations

This study has several limitations. First, the small sample size may restrict generalizability of our findings, reflecting the rarity of pediatric LMs and the challenges of recruiting a homogeneous cohort; however, strict eligibility criteria and standardized assessments helped reduce heterogeneity and support the reliability of the findings. Second, the 6-month follow-up period precludes the assessment of long-term effectiveness and recurrence. Third, the study was designed as an exploratory nonrandomized clinical trial and therefore provides only preliminary data on effectiveness and safety. Fourth, the study employed a full analysis set rather than a strict intention-to-treat approach. Finally, 1 patient lacked data after enrollment, which may have introduced some bias and renders both safety and effectiveness assessments unreliable.

## Conclusions

In this nonrandomized clinical trial of children with LMs, eppikajutsuto was associated with a reduction in lesion volume and was well tolerated. These findings suggest that eppikajutsuto may be effective in reducing LM volume, particularly in head and neck lesions, with a potentially favorable safety profile. In addition, eppikajutsuto may offer an alternative therapeutic approach with distinct mechanisms and tolerability advantages. Future studies should include functional and patient-reported measures although MRI-based volumetry provides objective outcomes. Further studies are also warranted to directly compare eppikajutsuto and sirolimus in randomized trials, to elucidate the mechanisms of action of eppikajutsuto, and to evaluate long-term outcomes through extended follow-up and multicenter studies. These steps are essential to fully establish the therapeutic role of eppikajutsuto in managing LMs.

## Notes

Supplement 1.
Trial Protocol and Statistical Analysis Plan

Supplement 2. **eTable.** Composition of Eppikajyutsuto Extract Granules

Supplement 3.
Data Sharing Statement
