# Supplementary material for: Eppikajutsuto for Treatment of Lymphatic Malformations in Children: A Nonrandomized Clinical Trial
Source: JAMA Netw Open. 2025 Nov 3;8(11):e2540897. doi: 10.1001/jamanetworkopen.2025.40897 (PMC12584033; doi:10.1001/jamanetworkopen.2025.40897)
Supplement: Supplement 1. — Trial Protocol and Statistical Analysis Plan [file jamanetwopen-e2540897-s001.pdf]

# Clinical Study to Evaluate the Effects of Eppikajutsuto on Lymphatic Vascular Malformations research plan

Abbreviated name: LMEP

Principal Investigator: Professor Keiko Ogawa, Center for Kampo Medicine, Hiroshima University Hospital
Address:〒 1-2-3 Kasumi, Minami-ku, Hiroshima 734-8551
Phone: 082-257-5461

Research Office: Center for Kampo Medicine, Hiroshima University Hospital
Chiyokori Maiiko
Address: 1-2-3 Kasumi, Minami-ku, Hiroshima 734-8551
Phone: 082-257-5461 FAX: 082-257-5461
E-mail: mako1125@hiroshima-u.ac.jp

April 9, 2021 Draft Plan Ver. 1.0
April 14, 2021 Draft Plan Ver. 1.1
June 1, 2021 Draft Plan Ver. 1.2
August 30, 2021 Plan Ver. 1.3
October 29, 2021 Plan Ver. 1.4
November 29, 2021 Plan Ver.1.5
January 12, 2022 Plan Ver.1.6
October 7, 2022 Plan Ver.1.7
October 7, 2022 Plan Ver.1.8
January 31, 2023 Plan Ver. 1.9
September 19, 2023 Plan Ver. 2.0
November 27, 2023 Plan Ver2.1
April 17, 2024 Plan Ver2.2

## Table of Contents


0. schema ..... 4
0.1. summary of clinical research design ..... 4
0.2. type of clinical research ..... 4
1. purpose ..... 4
2. background ..... 5
2.1. status of target diseases in Japan and abroad ..... 5
2.2. history and content of standard therapies implemented to date ..... 5
2.3. current standard of care and treatment results ..... 5
2.4. Issues, uncertainties, etc. in the current standard of care that lead to the need for such clinical research ..... 5
2.5. benefits and disadvantages of protocol treatment ..... 6
2.5.1. burdens and foreseeable risks to the research subjects ..... 6
2.5.2. anticipated benefits to the research subjects ..... 6
2.5.3. an overall assessment of these and measures to minimize burdens and risks ..... 6
2.6. Research Design ..... 7
3. eligibility criteria ..... 7
3.1. selection criteria ..... 7
3.2 Exclusion Criteria ..... 7
4. registration and allocation ..... 7
4.1. case registration procedure ..... 7
4.2 Allocation Method and Allocation Adjustment Factors ..... 8
5. clinical research plan ..... 8
5.1. Summary of Test Drugs ..... 8
5.2. medication group ..... 9
5.3. procedures and timelines over time for interventions such as medication, surgery, and laboratory tests ..... 9
5.3.1 Medication site, surgical site, examination site, etc. ..... 10
5.3.2 Timing and duration of interventions such as medication, surgery, tests, etc. ..... 10
5.3.3 Dosage, frequency, etc. ..... 10
5.3.4 Protocol treatment ..... 10
5.3.5 Dose and schedule change criteria ..... 10
5.4. adjunctive and supportive care ..... 10
5.5. post-treatment ..... 10
5.6. discontinuation of protocol treatment ..... 10
5.7 Control of Investigational Drugs, etc. ..... 11

6. observation, examination and reporting items and schedule ........ 11.
6.1 Observation and examination items and treatment information to be reported ........ 11
6.2. schedule of observations, inspections and reports ........ 12
6.3. preservation of samples, etc. and use of samples, etc. by other institutions, etc. ........ 12
7. target number of patients and duration of clinical study ........ 12
7.1. target number of cases ........ 12
7.2. duration of clinical research ........ 13
8. evaluation and reporting of adverse events (e.g., illness) ........ 13
8.1 Definition of Adverse Events (Diseases, etc.) ........ 13
8.2. evaluation and reporting of adverse events ........ 13
8.3. expected adverse events ........ 14
8.4. reporting and handling of serious adverse events ........ 14
8.4.1. reports of serious adverse events occurring in the conduct of the relevant clinical research (including serious adverse events resulting from malfunctions) ........ 14
8.4.2. report to the Minister of Health, Labor and Welfare, etc. ........ 15
8.4.3 Detailed and additional reports ........ 15
9. endpoint definition ........ 15
9.1. main endpoints ........ 16
9.2. secondary endpoints ........ 16
10. statistical considerations ........ 17
10.1 Basis for Setting Target Number of Cases ........ 17
10.1.1 Reestablishment of target number of cases ........ 17
10.2. population to be analyzed ........ 18
10.3 Analysis Items and Methods ........ 18
10.3.1. primary endpoint primary analysis method ........ 18
10.3.2. secondary analysis methods for primary endpoints ........ 18
10.3.3 Analysis Methods for Secondary Endpoints ........ 18
10.3.4. subgroup analysis ........ 19
10.3.5. significance level ........ 19
10.3.6 Handling of Missing Data, etc. ........ 19
10.4 Intermediate Analysis ........ 19
10.5 Procedures for modifying the statistical analysis plan ........ 19
11. completion and submission of case report form ........ 19
11.1. types and submission deadlines ........ 19
11.2. how to fill out the form ........ 20
11.3. method of delivery ........ 20

12. access to original documents, etc., and quality control and quality assurance ........ 20
12.1. acceptance of and cooperation with direct inspection ........ 20
12.2 Monitoring ........ 20
13. ethical matters ........ 21
13.1. rules and regulations to be observed ........ 21
13.2. review by an accredited clinical research review committee and notification to the administrator of the implementing medical institution regarding the commencement of research ........ 21
13.3. Preparation and Revision of Explanatory Documents and Consent Forms ........ 21
13.4. informed consent ........ 22
13.5. Consent by a Consenting Consenting Party ........ 23
14. handling of personal information ........ 23
15. deviations, changes or revisions to the research protocol ........ 24
15.1. deviations or changes in the research protocol ........ 24
15.2. approval and revision of research protocol ........ 24
16. termination and early termination of clinical studies ........ 24
16.1. termination of clinical research ........ 24
16.2. early termination of clinical research ........ 24
17. report to the administrator of the medical institution ........ 25
17.1. matters to be reported to the administrator of the executing medical institution ........ 25
17.2. matters to be reported to the Accredited Clinical Research Review Committee ........ 26
17.3. matters to be reported to the Minister of Health, Labour and Welfare ........ 26.
18. handling of materials related to clinical research ........ 26
19. payment of money and compensation for conducting clinical research ........ 27
19.1 Expenses for clinical research ........ 27
19.2. compensation for health hazards ........ 27
20. research funding and conflict of interest management ........ 27
20.1. conflict of interest management plan ........ 27
20.2. sources of research funding ........ 27
21. attribution of research results and publication of results ........ 28
22. research organization ........ 28
22.1. principal investigator ........ 28
22.2. principal investigator ........ 28
22.3 Image Evaluation Committee to determine effectiveness ........ 29
22.4. Research Secretariat (Coordination and Management Practitioner) ........ 29
22.5. person responsible for data management ........ 29
22.6. study statisticians and statistical analysts ........ 29

22.7. in charge of clinical epidemiology ........ 29
22.8. central monitoring officer ........ 29
22.9. Case Registry Center ........ 30
23 Complaints and Consultation ........ 30.
24. literature ........ 30
25 Appendix ........ 31

# 0. schema

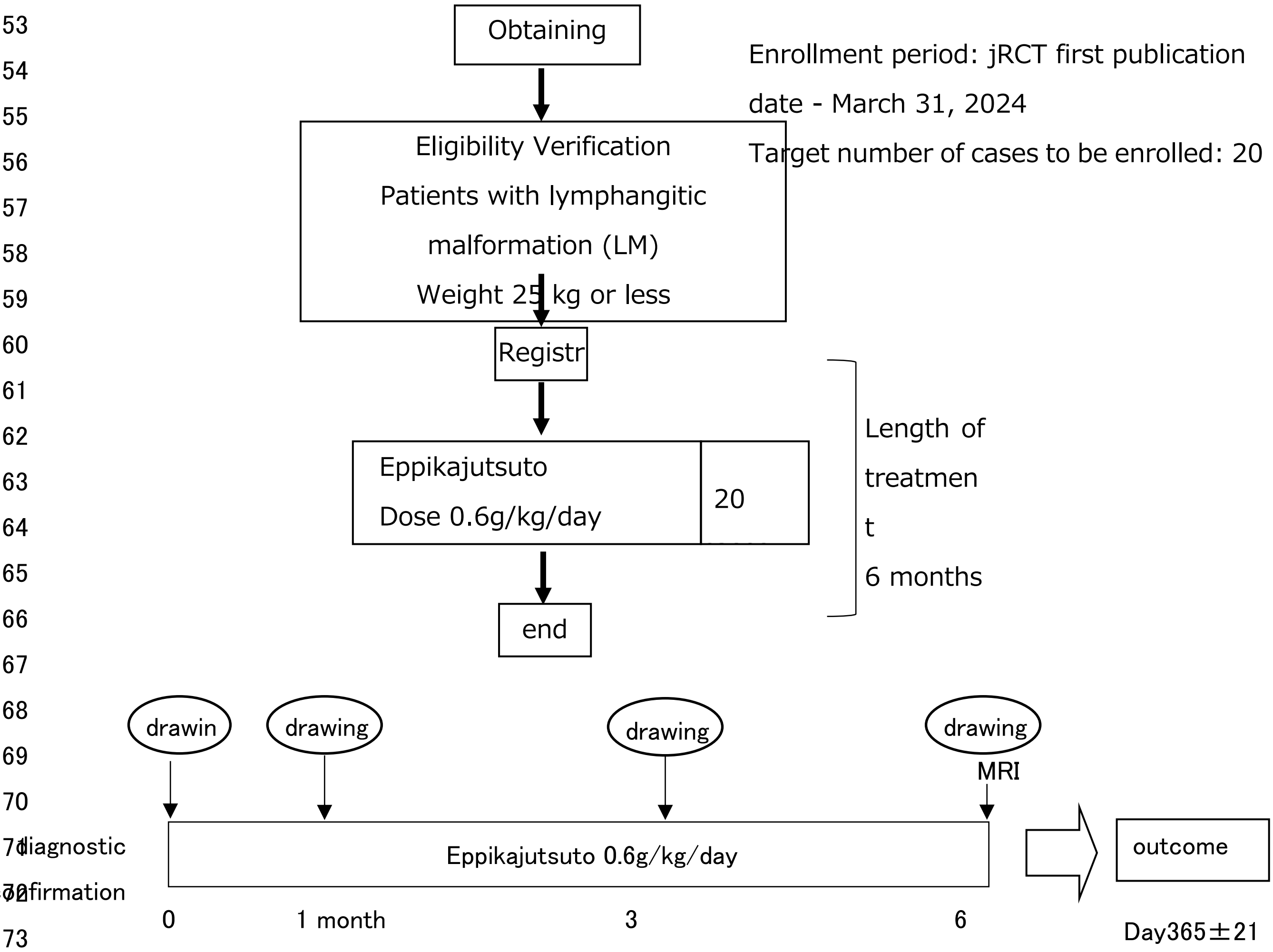


*If there are MRI images that meet the criteria within 3 months prior to registration, they should be used.

## 0.1. summary of clinical research design

Study Design: A prospective, interventional, Phase II, multicenter, open-label study with a pre- and post-treatment comparative design

## 0.2. type of clinical research

Type of clinical research: Specific clinical research (with unapproved or off-label use)

# 1. purpose

To evaluate the efficacy, dose-response, and safety of Eppikajutsuto in patients with lymphangitic malformations (LM) in an open-label, pre- and post-administration study. The primary endpoint will be response from baseline in tumor volume by volumetry. Secondary endpoints will be 1. 50% or greater volume reduction, 2. shrinkage rate, 3. 10% improvement in quality of life, 4. safety, and 5. adherence to medications.

# 2. background

## 2.1. status of target diseases in Japan and abroad

Lymphatic malformation (LM), a congenital malformation of the lymphatic system, occurs most often in the head and neck region and can cause significant complications such as bronchial compression, especially in children and neonates. LM is a chronic pediatric disease (lymphangioma), and under Japan's universal health insurance system, the main treatment modalities for LM are sclerotherapy and surgical resection. Sclerotherapy is particularly effective for cystic LM, but may be difficult to perform because it causes temporary swelling and compression of vital organs and tissues around the lesion. Surgical resection may also be difficult to implement in diffuse lesions where the lesions are not clearly limited and spread over a large area due to indistinct border growth. Resolution of these problems through less invasive treatment is desired. Recently, the general use of mTOR inhibitors such as sirolimus has been proposed for the treatment of LM in the United States and other countries. In Japan, sirolimus administration for LM is undergoing clinical trials.

The incidence of lymphangitic malformations (lymphangiomas) is unknown, but is estimated to be 1 in 1,000-5,000 births. The exact prevalence is unknown, but the estimated number of patients in Japan is about 10,000.([1)(2)]Spontaneous shrinkage is generally considered to be rare, and in two of the eight cases in our retrospective study in which we observed a natural history, the condition of LM was observed to be unchanging or worsening, with no spontaneous remission. The disease did not spontaneously remit in the two cases observed in our retrospective study.

## 2.2. history and content of standard therapies implemented to date

In Japan, the universal health insurance system mainly uses sclerotherapy and surgical resection for the treatment of LM, but less invasive treatments are desired. Recently, sirolimus has been approved in Europe and the United States and proposed as a common agent for LM, but potentially serious side effects have also been reported. Potentially serious side effects of sirolimus have also been reported, including thrombocytopenia, edema, anorexia, anemia, hyperglycemia, hyperlipidemia, hypertriglyceridemia, elevated alkaline phosphatase, elevated serum creatinine, lymphopenia, hypophosphatemia, infection and heart problems. In addition, the cost of continuous dosing of sirolimus is relatively high, at $^{3}$ 1,308.8/ mg, and prescriptions of 1 to 4 mg /day are common.

## 2.3. current standard of care and outcomes

As mentioned in 2.2, in Japan, the universal health insurance system provides sclerotherapy and surgical resection for the treatment of LM. According to the Guidelines for the Treatment of Hemangiomas, Vascular Malformations, and Lymphatic Malformations 2017[(1) ()], rated as strength of

recommendation: 2 (weak), across a wide range of treatment sites, treatment methods, age of treatment, and timing of treatment, the case-by-case outcomes for LM are not clear.

### 2.4. Issues, uncertainties, etc. in the current standard of care that lead to the need for such clinical research

While the standard therapies that have been implemented to date, as described above, are burdensome to patients in terms of side effects and cost, Eppikajutsuto has a high potential to solve these problems.

We were the first in Japan to report the effect of Echisukayaku-zhuyu on lesion reduction in mediastinal LM patients[(2)] [()] (Figure 1). Subsequently, many institutions have used Echisuyu-Kazhu-to, and in several retrospective case studies, we reported that reduction in mass size was observed in all 2, 8, and 9 cases, respectively[(3) (4) (4) (5) (5) (6))]. Based on these findings, it was inferred that an increased dose was necessary to achieve a reduction in size. Therefore, in this study, the dose was set at 0.6 g/kg/d in an exploratory manner. Although the insurance coverage for the use of extract is 7.5 g/d, we do not consider this to be particularly problematic, since the dosage is allowed to be increased as needed. The reason for this is that the subject of this study is limited to children, and the dose is doubled at 25 kg when the dose per body weight is doubled, but in this study, the dose is 7.5 g/day at 12.5 kg, so the dose was reduced at 12.5 kg or more. Therefore, the treatment above 12.5 kg can be regarded as treatment at a reduced dose, and it may be possible to evaluate this depending on the number of cases accumulated. There have been no reports of adverse drug reactions at doses such as 0.75 g/kg/d or 7.5 g/d for this dose[6)7)8].

We also reported an infant whose cervical LM was markedly reduced by an increased dose of Koshuizhuyu-tou. [9) ()]Furthermore, we also suggest an mTOR inhibitory effect by Koshui-Kazhu-tan in oral cancer cells.[(10) ()]

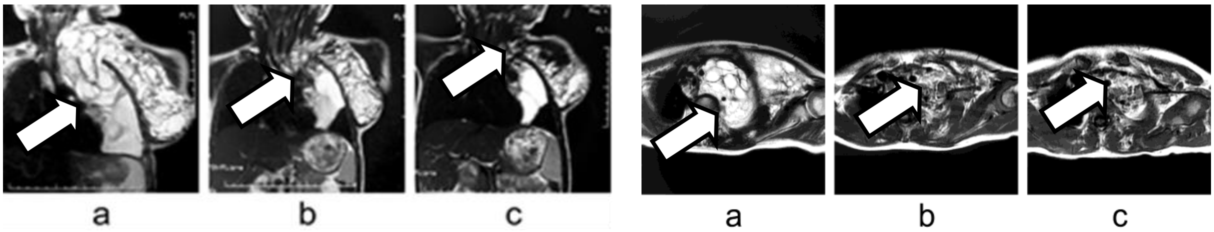


**Figure 1 Changes in mediastinal lymphatic malformations after administration of Chinese herbal medicine**

**a. Before administration b. After 6 months c. After 15 months**

Kesuyu-kazuto costs 11 yen/g, 0.3-0.7 g / kg / day for children, up to 7.5 g / day (= 82.5 yen), and is markedly less expensive than sirolimus.

On the safety front, according to product information from Tsumura Corporation, its side effects

include rash, nausea, anorexia, insomnia, palpitations, fatigue, and difficulty urinating, but the rate of side effects is very rare.

Although there are scattered reports on the efficacy of Eppikajutsuto for LM, no evidence has been established to evaluate its efficacy and its dose-dependence and safety.

## 2.5. benefits and disadvantages of protocol treatment

### 2.5.1. burdens and foreseeable risks to the research subjects

Participation in this study may result in adverse drug reactions.

### 2.5.2. anticipated benefits to the research subjects

The lesions may be improved by the effect of Kesuyu Kashuyu on LM.

### 2.5.3. an overall assessment of these and measures to minimize burden and risk

Evaluate as appropriate for anticipated burdens and risks, and monitor adverse events as appropriate to minimize risk.

## 2.6. Research Design

This clinical study will objectively establish the efficacy of Eppikajutsuto through an open-label pre- and post-administration comparative study and create evidence for combination therapy with Kampo medicine for LM.

We will prospectively compare the clinical efficacy and safety of Koshuizakazuto in a group of patients treated with LM. The rationale for this study is that Kesui-Saizhu-tou is used for LM patients, and there have been some cases of significant efficacy, so the study will not be conducted using placebo or a non-treated group as a control, but rather a before-and-after comparison between the same patients. In addition, the study will be conducted in a multicenter setting to minimize bias by institution.

# 3. Eligibility Criteria

## 3.1. selection criteria

1. patient with LM

（Specific cystic or spongiform lesions, at least on imaging (echo, CT, MRI)

2. weight 25 kg or less

## 3.2 Exclusion Criteria

A project shall be ineligible if one or more of the following items are met

1. patients with a history of allergic reactions to Koshuyuan

2. are receiving or have received mTOR inhibitors

3. who have undergone sclerotherapy or surgery in the past

4. who have serious diseases other than lymphatic malformations

5. patients who participated in other clinical studies (trials) within 3 months prior to the start of treatment with the study drug

6. patients for whom evaluable MRI cannot be performed

Other patients deemed inappropriate as research subjects by the principal investigator or subinvestigator

[Rationale] 1)-2)-3)Influence on efficacy evaluation and consideration for safety, 2)-3)Influence on efficacy evaluation, 4)-7)Consideration for safety

# 4. registration and allocation

## 4.1. case registration procedure

１）The principal investigator or subinvestigator obtains written consent from the research subject and the surrogate.

２）After obtaining consent, the principal investigator or subinvestigator will enter the necessary information (date consent was obtained, subject identification code, subject name, medical record number, etc.) that corresponds to the research subject and subject identification code in the list of research subjects kept by the principal investigator (or personal information manager, if a personal information manager is separately appointed). ), and the principal investigator (or personal information manager) will keep the list of research subject identification codes (or list of research subjects or register of research subjects) in a lockable place under his/her control.

３）After confirming that all selection criteria are met and none of the exclusion criteria are violated, the principal investigator or subinvestigator should complete the case registration form with the necessary information, including the subject identification code, and fax it to the case registration center.

４）The case registration center will verify eligibility and, if there are no eligibility issues, will prepare a registration confirmation form with the subject registration number and fax it to the principal investigator or research assistant.

５）After receiving and reviewing the registration confirmation form, the principal investigator or subinvestigator will initiate the protocol treatment.

６）In the event of withdrawal of consent, discontinuation, dropout, etc., the Principal Investigator shall be promptly informed.

## 4.2 Allocation Method and Allocation Adjustment Factors

No allocation

# 5. clinical research plan

## 5.1. Summary of Test Drugs

Test drug: Koshui-Kazuzu-to (product name: Tsumura Koshui-Kazuzu-to®, product number: 028)

Manufacturer (or distributor): Tsumura Corporation

Classification: Kampo formula

Indications: nephritis, nephrosis, beriberi, rheumatoid arthritis, nocturia, eczema

Route of administration, dosage, and directions for use (as indicated on the package insert):

The usual adult dosage is 7.5 g orally per day in two to three divided doses before or between meals. The dosage may be adjusted according to age, weight, and symptoms.

Contraindications: None

Main clinical use results: Action on dermatitis

Pre-mixing administration to mice suppressed primary irritant contact dermatitis caused by croton oil and allergic contact dermatitis caused by DNFB[(7) ()].

Side effects/defects:

●Serious side effects

Pseudohypoaldosteronism: Pseudohypoaldosteronism such as hypokalemia, elevated blood pressure, sodium and fluid retention, edema, and weight gain may occur.

Myopathy: Since myopathy may occur as a result of hypokalemia, patients should be carefully monitored. If abnormalities such as weakness, limb cramps or paralysis are observed, administration should be discontinued and appropriate measures such as administration of potassium agents should be taken.

Other side effects (frequency unknown)

Hypersensitivity: rash, redness, itching, etc.

Autonomic nervous system: insomnia, excessive sweating, tachycardia, palpitations, general weakness, mental agitation, etc.

Gastrointestinal: anorexia, gastric discomfort, nausea, vomiting, soft stool, diarrhea, etc.

Urinary organs: dysuria, etc.

Interactions: [Concomitant use precautionary statement].

The following drugs should be carefully administered: mallow-containing preparations, ephedrine-containing preparations, monoamine oxidase (MAO) inhibitors, thyroid preparations, thyroxine, liothyronine, catecholamine preparations, adrenaline, isoprenaline, xanthine preparations, theophylline, diprophyrin (since insomnia, excessive sweating, tachycardia, palpitations, general weakness, mental agitation, etc. are more likely to occur, the dosage should

be reduced or otherwise carefully administered), and other drugs. The dosage should be reduced or otherwise administered with caution since insomnia, excessive sweating, tachycardia, palpitations, general weakness, and mental agitation are more likely to occur.)

Preparations containing cambogia, glycyrrhizic acid and its salts (Pseudohyperaldosteronism is more likely to occur. Also, myopathy is more likely to occur as a result of hypokalemia.)

Usage Notes:

careful administration

1. Patients in the post-illness debilitated phase or those with significantly diminished physical strength [adverse reactions may be more likely to occur and symptoms may be intensified].
2. Patients with a weak gastrointestinal tract [Anorexia, gastric discomfort, nausea, vomiting, soft stools, diarrhea, etc. may occur.]
3. Patients with anorexia, nausea, and vomiting [these symptoms may worsen].
4. Patients with a marked tendency to perspire [excessive sweating, general weakness, etc. may occur.]
5. Patients with angina pectoris, myocardial infarction, or other circulatory disorders, or with a history of such disorders
6. Patients with severe hypertension
7. Patients with severe renal impairment
8. Patients with dysuria
9. Patients with hyperthyroidism [5-9: These diseases and symptoms may worsen.]

important basic precautions

When using this product, the patient's signs (constitution and symptoms) should be taken into consideration. The course of the patient should be carefully monitored, and if there is no improvement in symptoms and findings, continued administration should be avoided.

Since this drug contains cimpanzolamide, serum potassium and blood pressure levels should be carefully monitored, and administration should be discontinued if any abnormality is observed.

When other Kampo formulas, etc. are used concomitantly, care should be taken not to duplicate the herbal ingredients contained in the product.

See Appendix in "25. Appendix."

## 5.2. medication groups

All patients will be given medication.

## 5.3. procedures and timelines over time for interventions such as medication, surgery, and laboratory tests

The "study period" for each research subject will be from the date of obtaining consent to the date of completion of the outcome study in this study (Day365±21).

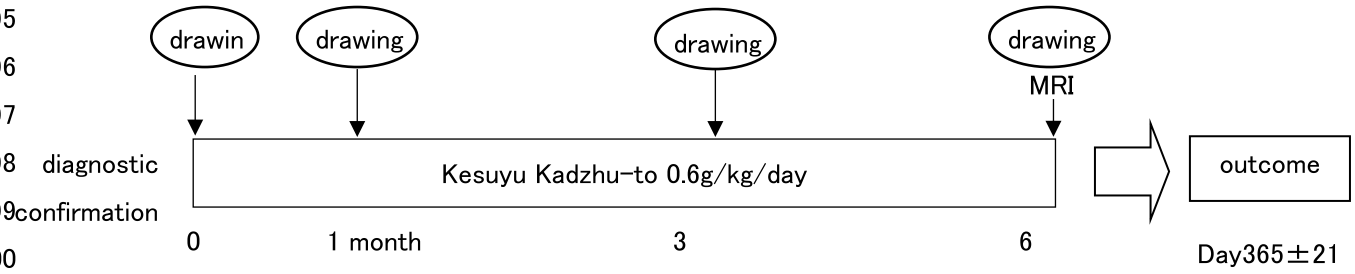


*If there are MRI images that meet the criteria within 3 months prior to registration, they should be used.

### 5.3.1 Medication site, surgical site, examination site, etc.

(doses of) oral administration

### 5.3.2 Timing and duration of interventions such as medication, surgery, tests, etc.

After consent is obtained, the study drug "Ketsuyu Kaju-to" will be taken internally for 6 months.

### 5.3.3 Dosage, frequency, etc.

The dosage is adjusted according to the body weight of the research subject. 0.6 g/min3 per kg of body weight per day of the test drug "Ketsuyu Kaju-to" is orally administered before meals. The dosage may be adjusted according to the increase or decrease of body weight. The maximum dosage is 7.5 g/day. The duration of treatment is 6 months.

### 5.3.4 Protocol treatment

Treatment (dosage, administration, and duration of administration)

All patients will be treated with the study drug (Kesuyu Kadzhu-to).

The dosage is adjusted according to the body weight of the research subject. 0.6 g/min3 per kg of body weight per day of the test drug "Tsumura Ketsuyu Kadzhu-to Extract" is orally administered before meals. The dosage may be adjusted according to the increase or decrease in body weight. The maximum dosage is 7.5 g/day.

The administration period will be 6 months after the start of the study treatment. Dosing should be initiated immediately after enrollment, and thereafter should be administered three times a day in conjunction with breakfast, lunch, and dinner (before and after meal times).

### 5.3.5 Dose and Schedule Change Criteria

Postponement: not administering at the prescribed date and time, but delaying it.

Postponements may be taken after a meal or at a later time if the dose is not taken at the predetermined time due to forgetting to take it or for other reasons.

Discontinuation: permanent and continuous withdrawal of the entire treatment or a specific drug.

It is assumed that the drug will not be restarted.

Keep a record of the patient's medication intake and monitor the patient's medication status. If adherence is poor and the patient is unable to take the medication, discontinue the medication. In such a case, continue to monitor the patient.

## 5.4 Adjunctive and Supportive Therapy

Sclerotherapy, surgical resection, and concomitant use of mTOR inhibitors such as sirolimus and laparolimus are prohibited.

## 5.5. post-treatment

After discontinuation of protocol treatment or completion of protocol treatment, post-treatment is not restricted.

## 5.6. discontinuation of protocol treatment

The investigator or subinvestigator will discontinue the study drug if the investigator or subinvestigator determines that continuation of protocol treatment is not feasible for any of the following reasons. The date, reason, and progress of discontinuation will be documented in the medical record and the case report form (CRF), and necessary tests will be performed at the time of discontinuation or dropout to evaluate efficacy and safety.

If the drug is discontinued due to the occurrence of an adverse event, the patient should be followed up as much as possible until the current status is restored.

(1) When a research subject or a surrogate withdraws from participation in clinical research or withdraws his/her consent.

(2) When it is found that eligibility is not satisfied after registration

(iii) When the underlying disease is completely cured and there is no longer a need for continued administration

(iv) When it is determined that continued administration of the test drug is not desirable due to worsening of the underlying disease

(5) When it is difficult to continue the clinical research due to exacerbation of complications

(6) When it is difficult to continue the clinical research due to adverse events

(vii) In case of significant non-adherence (when it is determined that less than 70% of the total scheduled doses will be taken or more than 120% of the total scheduled doses will be taken)

(viii) If the research subject does not come to the hospital due to relocation, etc.

(ix) When the entire clinical research is discontinued

(10) When the principal investigator determines that it is appropriate to discontinue the clinical research for other reasons

## 5.7 Control of Investigational Drugs, etc.

The study drug custodian will manage the provided study drug, residual study drug, and disbursement of the study drug. The test drug(s) shall be used only for this study and shall not be used for any other purpose.

To maintain the quality of the test drug, it should be stored in a cool place out of direct sunlight and away from moisture as much as possible.

# 6. observation, examination, and reporting items and schedule

## 6.1. observation and examination items and treatment information to be reported

●before the start of the test

Research subject information: identification code, gender, date of birth, height, weight, complications, pre-existing medical conditions, current medical history, previous treatment, and allergies.

Blood tests: blood count, platelets, peripheral hemogram

Blood biochemical tests: BUN, Cr, T-Bil, AST-GOT, ALT-GPT, LDH,γGTP, Na, K, Cl, T-Chol, CRP

Diagnostic Imaging (MRI)

●Day28 (Tolerance: Day28±3), Day84 (Tolerance: Day84±7)

Blood tests: blood count, platelets, peripheral hemogram

Blood biochemical tests: BUN, Cr, T-Bil, AST-GOT, ALT-GPT, LDH,γGTP, Na, K, Cl, T-Chol, CRP

●Day 168 (Tolerance: Day 168 ± 14)

Blood tests: blood count, platelets, peripheral hemogram

Blood biochemical tests: BUN, Cr, T-Bil, AST-GOT, ALT-GPT, LDH,γGTP, Na, K, Cl, T-Chol, CRP

Diagnostic Imaging (MRI)

●Outcome study (Day365±21)

The course of symptoms will be followed, including the frequency of requests for continued use of Eppikajutsuto after completion of protocol treatment and a subsequent survey of medical records.

●Confirmation of medication adherence

●QOL questionnaire survey by EQ-5D-5L Japanese version for Japan.

## 6.2. schedule for observation, inspection and reporting

Observation, Inspection and Reporting Schedule

| (data) item | Consent and screening | protocol treatment | outcome study | time of discontinuance |
|---|---|---|---|---|
| time | before the start of the test | Day 0 Day28 Day 84 Day168 | Day365 | Date of Decision to Cancel |
| Obtaining Consent | ○ | | | |
| Confirmation of medication adherence | | ○ ○ ○ | | |
| Background check | ○ | | | |
| test drug administration | | ◀———————▶ | | |
| Observation of adverse events | ○ | ◀———————▶ | | |
| clinical examination: hematology<br>blood chemistry | | ○ ○ ○ ○<br>○ ○ ○ ○ | △ | ○<br>○ |
| MRI | ○note (supplementary information) symbol | ○ | △ | ○ |
| EQ-5D-5L | | O ○ | | |

△Implementation: Implemented when possible

*If there are MRI images that meet the criteria within 3 months prior to registration, they should be used.

## 6.3. preservation of samples, etc. and use of samples, etc. by other institutions, etc.

MRI imaging data, medical data, and blood test data will be provided to the investigators for before and after comparison of the effects of the study drug. The principal investigator will instruct the subinvestigators to properly store the samples in accordance with the established storage methods, and will take necessary measures to prevent leakage, mixing, theft, or loss of the samples. The amount of blood collected should be between 3 ml and 10 ml depending on the method of blood collection, and the collected serum or plasma should be stored frozen at each medical institution until March 31, 2028 after the completion of the clinical research. When disposing of the samples, they will be anonymized and personal information will be carefully handled.

Samples and information of research subjects obtained in such clinical research may be used for future research that is not identified at the time consent is obtained. In such a case, the research will

be conducted only after the implementation of the research based on the samples obtained in the relevant clinical research is reviewed by an ethical review committee in accordance with the content of the research and is separately explained to the research subject and the surrogate consentor.

# 7. target number of patients and duration of clinical study

## 7.1. target number of cases

Overall clinical research: Number of participating facilities: 7, Target number of cases: 20

| Name of medical institution | Target number of cases |
|---|---|
| Hiroshima University | 3 |
| Kanazawa University | 4 |
| Osaka University | 3 |
| Nihon University | 3 |
| National Center for Child Health and Development | 3 |
| St. Marianna Medical University | 2 |
| Showa University Hospital | 2 |

## 7.2. duration of clinical research

Registration period jRCT first publication date - March 31, 2024

Period of the study jRCT first publication date - March 31, 2026 (from registration of the implementation plan in the Japan Registry of Clinical trials (jRCT) to registration of the summary report summary in the jRCT)

# 8. evaluation and reporting of adverse events (e.g., illness)

## 8.1 Definition of Adverse Events (Diseases, etc.)

Adverse Event (Disease, etc.) means all unwanted or unintended injuries or illnesses or signs thereof (including abnormal laboratory values) that occur in a Research Subject, regardless of whether or not they are causally related to the clinical research conducted. (2) "Unintended injury or disease" means any unfavorable or unintended injury or disease or its symptoms (including abnormalities in clinical laboratory values) that occurs in research subjects, whether or not there is a causal relationship with the clinical research conducted. In this clinical research, all undesirable or unintended injuries or illnesses, or signs thereof (including abnormal laboratory values) that occur in research subjects after the administration of Kesuyu Kashuyu is started shall be considered as adverse events. Adverse Events.

Serious adverse event" means an adverse event that falls under any of the following categories

1) Deadly

2) May lead to death

3) Admission to a medical institution for treatment or prolonged hospitalization is required

4) Obstacles

5) May lead to impairment

6) Congenital diseases or anomalies in later generations

The term "unanticipated serious adverse event" means a serious adverse event that is not described in the research protocol, informed consent document, etc. or, even if described, the nature or severity of the event is not consistent with the description.

An adverse drug reaction (ADR: adverse drug reaction) is an adverse event for which a causal relationship to the use of the drug in question cannot be denied (including those for which the causal relationship is unknown).

## 8.2. evaluation and reporting of adverse events

In this clinical study, adverse events and side effects will be evaluated using CTCAE v4.0-JCOG, with adverse events listed and graded. Grading of adverse events will be based on the closest definition of Grade 1 to 4, respectively. If a specific treatment is listed in the Grade, it should be graded based on its clinical necessity. In this study, an adverse event is judged as a Grade worsening by one or more levels in CTCAEv4.0. Abnormal laboratory values are also graded in the same way. Adverse events that occurred prior to treatment with the study drug will also be reported as adverse events if the Grade worsens by one or more levels.

Severity of adverse events

The severity of the adverse event shall be one of the following

1. non-serious

2. serious

Causality Classification

The causal relationship to the study drug and protocol treatment will be determined by one of the following categories, respectively

1. causality cannot be ruled out: there is a reasonable causal relationship with the study drug or protocol treatment (including cases where causality is unknown due to insufficient information)

2. causality can be ruled out: there is no reasonable causal relationship to the study drug or protocol treatment.

Adverse Event Outcomes

Adverse event outcomes shall be classified as one of the following categories.

1. recovery: adverse event returned to Grade 0 in CTCAE v4.0 or Grade prior to study drug administration

2. minor: adverse event returned to Grade 1 in CTCAE v4.0 (applies to AEs Grade 2 or higher)

3. unrecovered: Adverse event has not resolved and is in the same Grade as when the adverse event occurred.

4. recovered but with sequelae: adverse events resolved but with sequelae

5. death: The research subject has died

6. unknown: no information, unknown outcome

Response to adverse events

When an adverse event occurs in a research subject, the principal investigator and the research assistant investigator will first ensure the treatment and safety of the research subject and take appropriate measures. The observation period for adverse events in this clinical research will be from the start of protocol treatment until 4 weeks after the protocol treatment is completed or discontinued, and follow-up observation will be conducted thereafter until recovery as much as possible. However, this does not apply to cases in which symptoms that develop due to worsening of the primary disease or complications of the research subject have become chronic, or in cases in which continuous observation is difficult due to transfer to a different hospital or start of post-treatment.

The principal investigator and the research assistant investigator will make the following evaluations of the adverse events that occur and document them in the case report form. If more than one adverse event occurs, the adverse event should be judged separately.

1. identification of suspect drug

2. severity and severity of adverse events that occurred

3. existence of a causal relationship with the study drug

4. predictability

When a serious adverse event is recognized, the principal investigator shall promptly report it to the head of the institution in accordance with the institution's procedures, as well as to the principal investigators at other institutions.

Once a year, the principal investigator reports the progress of the clinical research and the occurrence of adverse events associated with the implementation of the clinical research to the Accredited Clinical Research Review Committee and the administrator of the medical institution where the clinical research is conducted.

### 8.3. anticipated adverse events

Serious Side Effects of Kesuyu Kashuyu

1) Pseudohyperaldosteronism: frequency unknown 2) Myopathy: frequency unknown

### 8.4. reporting and handling of serious adverse events

The Principal Investigator will make arrangements, including a contract/memorandum of understanding (MOU), with the study drug provider, Tsumura Corporation, prior to the start of the

study regarding adverse event reporting procedures and reporting details. Communication with the principal investigator and other relevant personnel at each research institution will be in accordance with the attached "Procedures for handling illnesses and other incidents in clinical research to evaluate the efficacy of Eppikajutsuto for lymphatic malformations".

#### 8.4.1. report of serious adverse events occurring in the conduct of the clinical research (including serious adverse events resulting from malfunctions )

Reports should be made in accordance with the "Procedures for Reporting Diseases and Malfunctions in Clinical Research under the Clinical Research Act" or other similar procedures at each site.

1) In the event of a serious adverse event (including serious adverse events that may occur due to malfunction), the research physician shall take appropriate measures and immediately report to the principal investigator, regardless of the causal relationship to the study drug.
2) The investigator shall promptly determine whether or not the serious adverse event has a causal relationship to the conduct of the clinical research and whether or not it is a known event, and respond to it in accordance with the "Procedures for Reporting Diseases and Defects in Clinical Research under the Clinical Research Act at Kanazawa University". That is, in the event of a serious adverse event, a "Report of Disease, etc. (First Report) (Uniform Form 8)" (and a "Report of Disease, etc. (Pharmaceuticals)" (Appendix Form 2-1) in the case of a serious adverse event requiring a report to the Minister of Health, Labor and Welfare) shall be filed and reported to the hospital director within 72 hours from the time of knowledge (for events that have a suspected causal relationship with the relevant clinical research). (The secretariat of the Accredited Clinical Research Review Committee will also be submitted for events that are suspected to have a causal relationship with the clinical research in question).
3) The Research Office will report the details reported in 2) above to the administrator of Kanazawa University Hospital, the Principal Investigator, other investigators, and the manufacturer and distributor of the study drug, as well as to the Effectiveness and Safety Evaluation Committee as necessary.
4) Other principal investigators report to the administrator of the medical institution to which they belong.
5) The administrator of the site will take necessary measures for the adverse event.
6) The secretariat of the Accredited Clinical Research Review Committee shall report the opinions of the Accredited Clinical Research Review Committee to the Research Office, which in turn shall report the contents of the report to the administrator of Kanazawa University Hospital, the principal investigator, the principal investigator and the manufacturer and seller of the study drug.

7) Details of further procedures regarding adverse events will be in accordance with the serious adverse event procedures established by each institution.

<Contact information in case of emergency

Center for Chinese Medicine, Hiroshima University Hospital
Address: 1-2-3 Kasumi, Minami-ku, Hiroshima City
Phone: 082-257-5461
FAX : 082-257-5461

### 8.4.2. report to the Minister of Health, Labor and Welfare, etc.

In the event of an unanticipated serious adverse event suspected to have resulted from the conduct of said clinical research, the investigator shall submit a "Report on Disease (Pharmaceuticals)" (Appendix Form 2-1) to the Minister of Health, Labour and Welfare within 7 days of learning of the occurrence regarding death or the threat of death, and within 15 days otherwise.

### 8.4.3 Detailed and additional reports

For serious adverse events reported to an accredited clinical research review committee, the investigator shall submit a "Report of Drug Disease, etc." (and a follow-up report of the "Report of Disease, etc." if necessary) to the accredited clinical research review committee.

If the outcome of such adverse event is "unrecovered" or "unknown," the patient should be followed up as much as possible.

# 9. endpoint definition

## 9.1. main endpoints

Primary Endpoint: Tumor response assessed by MRI-based volumetric analysis at 6 months, defined as at least a 20% reduction in volume. For diffuse lesions, reduction in the primary lesion size will be assessed.

Rationale: Based on past case series, a measurable degree of shrinkage is expected at 6 months of drug administration.

Prior to lesion evaluation, a definitive diagnosis is made by the imaging evaluation committee to determine efficacy.

Methods of measuring lesions

1. MRI is performed.

Required imaging methods:

T1WI; T1-weighted image, T2WI; T2-weighted image, T2FS; T2 fat suppressed image, 3D-T2FS; 3D-T2 fat suppressed image. In all cases, axial sectioning is mandatory.

Gd-T1FS; post-contrast T1 fat suppression images are not required and may be taken only when necessary for differential diagnosis.

T2 fat-suppressed images should be taken in gapless mode. The basic slice thickness is 3-5 mm, but if the lesion is large, 6-8 mm is acceptable.

Definitive evaluation is based on volume comparison, using 3D-T2FS, or if 3D-T2FS is not available, T2FS with gapless imaging.

2. evaluation method

Two evaluators (Imaging Evaluation Committee: Diagnostic Radiologist) who meet the following criteria

(1) be an external evaluator, and (2) be a central diagnosis rather than a facility-by-facility evaluation.

OsiriX, a DICOM viewer, shall be used for measurement.

Before evaluation, the evaluators confirm the extent of the lesion to be evaluated in two or more directions, such as axial and coronal sections, and establish the appropriate measurement area. As a rule, the volume is measured using the axial section image.

For the measurement, two evaluators shall independently measure the volume. The measured values shall be rounded off to two decimal places. The average of the two evaluators' final evaluation values shall be the final evaluation value. The inter-observer correlation coefficient (ICC) is calculated to evaluate the reproducibility of the measurement values among the evaluators.

If the respective values differ by more than 10%, it is desirable to confirm the ranges measured by each other, since the ranges and sites to be evaluated may be very different.

After the measurement, all the area and ROI information measured in each slice should be saved as the original data of the measured values. Specifically, the data should be saved in a movie, JPEG image, or other format that separates the evaluation period for each patient, and submitted to the secretariat together with the data of the definitive evaluation value.

The primary evaluation is a comparison of pre-treatment and 6 months post-treatment.

3. measurement area

The volume of the lymphatic malformation lesion will be measured objectively for the target lesion determined at the start of the study.

## 9.2. secondary endpoints

1. percentage decrease of more than 50% of volume
2. lesion reduction rate
3. 10% improvement in quality of life associated with treatment
4. safety of administration of koshuizhuyuu
5. adherence to the administration of Koshuizhuyuu (percentage of patients who were able to take

at least 70% of their medications)

Rationale: 3) Even if there is no marked change in size, QOL and symptom improvement has been reported, so it was set as a secondary endpoint; 4) Pseudohyperaldosteronism is known to be a major side effect of Eppikajutsuto, but its occurrence in children is not well reported.

# 10. statistical considerations

## 10.1 Basis for Setting Target Number of Cases

In our retrospective case series study, the response rate (20% volume reduction at 6 months) to treatment with Eppikajutsuto in LM patients was 83% (in submission). Conservatively taking into account the overestimation tendency of the retrospective study design of prior reports, we expect a response rate of at least 50% with this treatment in the study's patient population. In the same study, none of the patients had a response to no treatment, and both patients had an increasing trend. Considering the exploratory nature of this study, the first prospective clinical trial, the number of patients will be designed based on accuracy. The lower limit of the two-sided 90% confidence interval (corresponding to a one-sided significance level of 5%) is 29.1% (the upper limit is 70.8%) if 9 patients respond (response rate of 50%) in the 18 patients in the analysis.

Considering that the main purpose of this exploratory study is to collect information that can be used for the next phase of the study, we believe that 18 cases is an appropriate number of cases. Since the number of patients seen annually at each site is approximately 5 cases each, it is possible to enroll the target number of cases in 3 years.

Based on the above considerations, the target number of cases was set at 20 to ensure accuracy in case a few cases were excluded from the analysis.

### 10.1.1 Reestablishment of target number of cases

As mentioned in 10.1, considering the rarity of the subject cases in this study, we believe that the number of cases of 20 is close to the upper limit from the viewpoint of feasibility. On the other hand, considering that the main purpose of this study is to collect information that can be used for the next phase, although it is positioned as an exploratory study, the collection of exploratory data that contributes to the identification of subpopulations with higher potential for efficacy is one of its important objectives, and a larger number of cases would be preferable as long as feasibility permits.

Based on the above considerations, if it becomes clear during the study period that the target number of 20 cases can be enrolled earlier than previously expected, the target number of cases will be considered for re-setting according to the progress at that point in time. From the viewpoint of feasibility, we currently plan to slightly increase the target number of patients at that time as well. However, we will consider re-setting the target number of patients appropriately according to the information that is updated and accumulated both inside and outside the study up to that point.

## 10.2. population to be analyzed

The primary analysis of the primary and secondary endpoints will be performed on the Full Analysis Set (FAS). Analysis will also be performed on the Per Protocol Set (PPS) and compared with the FAS to confirm the stability and sensitivity of the analysis results.

The FAS is defined as a population that excludes from all enrolled cases any case that falls into one of the following categories: (1) cases with a confirmed diagnosis of a disease that is not eligible, (2) cases that violate important exclusion criteria, (3) cases that have never received protocol treatment since enrollment, or (4) cases with no data at all since enrollment.

The PPS will be defined as patients who meet the minimum requirements for key variables in the study protocol, for whom protocol treatment was not possible or was discontinued, but who have completed MRI evaluation and have no serious protocol violations regarding eligibility criteria, concomitant prohibited drugs, etc. The Safety Analysis Set (SAS) is defined as all enrolled patients, excluding those who have never received protocol treatment since enrollment.

## 10.3 Analysis Items and Methods

### 10.3.1. primary endpoint primary analysis method

For the primary endpoint of the study, the response rate will be based on whether or not patients achieve a reduction in lymphatic malformation volume by volumetry of at least 20% from the pre-treatment level at 6 months of treatment with the drug. An exact method based on a binomial distribution will be used for interval estimation. The significance level for the main analysis is 5% one-sided, and the corresponding two-sided 90% confidence interval is calculated.

### 10.3.2. secondary analysis methods for the primary endpoint

For reference, the same analysis as in 10.3.1 will be performed with the PPS as the population to be analyzed.

### 10.3.3 Analysis Methods for Secondary Endpoints

1. reduction of more than 50% of the volume

   For each case, the presence or absence of a reduction of 50% or more in the volume of lymphatic malformations by volumetry at 6 months of drug administration from before the start of treatment is determined, and based on this, the percentage of volume reduction of 50% or more is determined. For interval estimation, an exact method based on binomial distribution is used and two-sided 95% confidence intervals are calculated.

2. shrinkage rate

For each case, determine the percentage reduction in volume of lymphatic malformations by volumetry at 6 months of drug administration from before the start of drug administration.

3. 10% improvement in QOL

Use EQ-5D-5L Japanese version for Japan.

Depending on the respondents' understanding of the questionnaire, Effective_Japan (Japanese) EQ-5D-5L Paper Self-Complete v1.2 or Effective_Japan (Japanese) EQ-5D-5L Paper Proxy2 v1.1 is used to determine the percentage of improvement in quality of life by 10% or more.

4. safety

For adverse events, determine the type and frequency along with a list. If necessary, use an accurate method based on a binomial distribution when determining confidence intervals .

5. medication adherence

Obtain numerical values for medication adherence. If necessary, use an exact method based on a binomial distribution when determining confidence intervals.

### 10.3.4 Subgroup Analysis

Obtain response rates as in 10.3.1 for each of the following subgroups.

- gender
- Site of lesion
- Medication adherence

Age

- Nature of lesion (cystic lesions are defined as cysts >2 cm in diameter)

### 10.3.5. significance level

Unless otherwise noted, the significance level is 5% one-sided, and the confidence coefficient for the confidence interval is correspondingly 90% two-sided.

### 10.3.6 Handling of Missing Data, etc.

Missing values are not compensated for, and unless otherwise noted as the analysis method, the analysis is performed without using the completion method.

## 10.4 Intermediate Analysis

No intermediate analysis is performed.

### 10.5 Procedures for modifying the statistical analysis plan

Changes to the statistical analysis plan shall be implemented upon agreement between the principal investigator and the study statistician or other relevant personnel. Changes to the statistical analysis plan must be made prior to conducting the relevant analyses affected by the change. Changes that affect the entire research plan must be implemented in accordance with "15.2. Approval and revision of research protocol".

## 11. completion and submission of case report form

### 11.1. types and submission deadlines

The types of CRFs to be used in the clinical research and the submission deadlines are as follows

| | |
|---|---|
| (1) Registration Eligibility Verification Form | at registration |
| (2) Pre-treatment, progress, and end of treatment | Discontinue protocol treatment/as soon as possible |
| (3) Follow-up and outcome studies | Within the timeframe indicated on the survey form |

The principal investigator or subinvestigator should prepare the case report form in accordance with the "Guidance for Preparation, Modification, or Amendment of Case Report Forms" . The case report form may be completed by the research collaborator under the supervision of the principal investigator or subinvestigator. However, items that do not involve medical judgment may be completed by the research collaborator under the supervision of the principal investigator or research collaborator. The principal investigator will confirm that there are no problems with the contents of the case report forms prepared by the subinvestigator or research collaborator. The principal investigator submits the completed case report form to the data center.

The case report should include the following statements

- Obtaining Consent
- Background of Study Subjects
- inspection results
- Information on administration of study drug, etc.
- Information on concomitant treatment
- Adverse Event Information

### 11.2. how to fill out the form

The principal investigator or subinvestigator will promptly prepare the case report form. Changes or modifications to the case report form should be made in accordance with the "Guidance for Preparation, Changes, or Modifications to Case Report Forms. The principal investigator will also inspect any changes or modifications to the case report form made by the subinvestigators to ensure

that there are no problems. If there are any discrepancies between the entries in the case report form and the source documents, the principal investigator will prepare and maintain a record explaining the reasons for the discrepancies. If an inquiry is made regarding the contents of the case report, the investigator will promptly respond to the inquiry, and if any modification is necessary, the investigator will take appropriate action.

### 11.3. method of delivery

To the data center, by fax.

Medical Research Support
〒541-0043
6F Koraibashi Yamamoto Building, 3-1-14 Koraibashi, Chuo-ku, Osaka
TEL: 06-6202-5444
fax: 06-6202-5445

To avoid the risk of disclosure of research subject personal information, use the research subject registration number and not the institution's medical record number when contacting the data center, such as for requests to send CRFs.

## 12. access to original documents, etc., and quality control and quality assurance

### 12.1. acceptance of and cooperation with direct inspection

To ensure the credibility of the study, the Monitoring Officer, the Clinical Research Review Committee, and the regulatory authorities may conduct direct inspection of case data and documents pertaining to the conduct of the study.

### 12.2 Monitoring

The principal investigator will develop a monitoring protocol. The principal investigator will designate a person to engage in monitoring. The principal investigator or monitoring staff will prepare a monitoring plan. Based on the monitoring protocol and monitoring plan, the monitoring staff will monitor, through central monitoring, whether the clinical research is being conducted in accordance with regulatory requirements and the relevant research protocol. The monitoring staff will prepare a monitoring report after monitoring and promptly submit it to the principal investigator. The principal investigator will provide information on the report to the other principal investigators.

- Are the human rights of subjects of clinical research protected and their safety ensured?
- Is the clinical research being conducted in compliance with the latest implementation plan, research protocol, and these rules and regulations?
- Has written consent for conducting clinical research been obtained from the subject of the

clinical research?

- Are records and other documents accurate?

Monitoring will be conducted as soon as possible after enrollment of the first patient, after completion of protocol treatment of the first patient, and after completion of protocol treatment of the last patient enrolled. If any problems are found, the monitoring frequency will be increased. The monitoring staff will prepare a monitoring report and submit it to the principal investigator as soon as possible after the monitoring is conducted. The monitoring report should include the date of monitoring, materials reviewed, the person conducting the monitoring, and a summary of any significant findings or facts, such as disease or noncompliance. The principal investigator will notify the principal investigator of the monitoring results as necessary (notification to the principal investigator is mandatory when significant findings such as disease or nonconformity are reported). Upon receipt of notification, the principal investigator will provide information on the notification to other principal investigators.

## 12.3 Auditing

The principal investigator will designate a person to conduct the audit. The person engaged in the audit will conduct the audit by directly inspecting the source documents from the following perspectives

- Whether the clinical research is being conducted in compliance with the latest implementation plan, research protocol and procedures (including reporting and monitoring of diseases, etc.) and these rules

- Are records and other documents accurate?

As for the frequency of conducting the audit, it shall be conducted promptly after the completion of protocol treatment and after the collection of the CRF of the final enrollee. The site to be audited after completion of protocol treatment and collection of the CRF of the last enrollee will be designated by the Principal Investigator, taking into consideration the number of enrolled research subjects, the occurrence of serious adverse events, and deviations based on monitoring reports. The auditor will prepare an audit report promptly after the audit and submit it to the principal investigator. A copy of the audit report shall also be submitted to the principal investigator of the institution to be audited. The audit report should include the date of the audit, the source documents reviewed, the person conducting the audit, and a summary of the contents of any significant findings or facts, such as diseases, noncompliance, etc.

# 13. ethical matters

## 13.1. rules and regulations to be observed

All persons involved in the said clinical research shall carefully read and understand the contents of the "Declaration of Helsinki of the World Medical Association" and the Clinical Research Act and related ministerial ordinances and notifications, etc., to which all medical research involving human subjects should conform, and shall comply with them. The implementation plan for this study, which was approved by an accredited clinical research review committee, has been published in jRCT.

## 13.2. review by an accredited clinical research review committee and notification to the administrator of the implementing medical institution regarding the commencement of research

Before conducting the said clinical research, the principal investigator shall submit the said research protocol and implementation plan, explanation and consent documents, documents describing the outline of drugs, etc., procedure manuals for dealing with the occurrence of illnesses, etc. suspected to be caused by the implementation of the said clinical research, monitoring procedure manuals, and other documents required by the accredited clinical research review committee, to the accredited clinical research review committee for its opinion. The principal investigator and investigator-in-charge shall submit the documents outlined in the summary of the drug, etc., the procedure for responding to the occurrence of a disease, etc. suspected to be caused by the implementation of the clinical research, the monitoring procedure, and other documents required by the Authorized Clinical Research Review Committee, and hear their opinions. The Principal Investigator and Responsible Investigator shall submit the documents submitted to the Accredited Clinical Research Review Committee and the opinions of the Accredited Clinical Research Review Committee to the administrator of the implementing medical institution to obtain approval to conduct the clinical research, and then commence the clinical research.

## 13.3. Preparation and Revision of Explanatory Documents and Consent Forms

The investigator shall prepare the explanatory and consent documents and revise them as necessary. The prepared or revised explanation and consent documents should be submitted to an accredited clinical research review committee in advance. The administrator of the institution should be notified of the revised documents together with the opinion of the Accredited Clinical Research Review Committee, and approval should be obtained.

The explanatory document must contain at least the items specified in the "Enforcement Regulations of the Clinical Research Act" as follows. The description must not intentionally induce research subjects. In the case of a multicenter form, common items other than those specific to each institution (e.g., the name of the principal investigator and contact information for

the consultation service) should be listed so that the contents of the description regarding the explanation to the subjects of clinical research and their consent at each institution match when submitted to the authorized clinical research review committee.

- The name of the specific clinical research to be conducted, a statement that approval has been obtained from the administrator of the medical institution conducting the specific clinical research, and a statement that an implementation plan has been submitted to the Minister of Health, Labour and Welfare.
- Name of the implementing medical institution and the name and title of the principal investigator (when the specified clinical research is conducted as a multicenter joint research, the name and title of the principal investigator and the name of other implementing medical institutions, including the name and title of the principal investigator of such institutions).
- Reason for selection as a subject for specific clinical research
- Anticipated benefits and disadvantages of conducting specific clinical research
- that refusal to participate in a specific clinical research study is voluntary.
- Matters concerning withdrawal of consent
- That no one will be treated unfavorably for refusing to participate in the specified clinical research or for withdrawing consent.
- Regarding the handling of data in the event of discontinuation, a statement that results obtained up to the announcement of withdrawal of consent will be used only after obtaining renewed consent.
- Methods of Information Disclosure Regarding Specific Clinical Research
- (2) A statement that the research protocol and other materials relating to the conduct of the Specified Clinical Research may be obtained or inspected at the request of the subject of the Specified Clinical Research or the subject's representative, and the method of obtaining or inspecting such materials.
- Matters Concerning Protection of Personal Information of Subjects of Specific Clinical Research
- Methods of storage and disposal of samples, etc.
- (iii) Status of provision of research funds, etc. and other involvement by the manufacturer or distributor of the drug, etc. in the specified clinical research, and status of provision of remuneration and other involvement by the manufacturer or distributor of the drug, etc. for contributions, manuscript writing, lectures, and other services with respect to persons engaged in clinical research and persons listed in the research protocol.
- System for handling complaints and inquiries
- Matters related to the costs associated with the implementation of specific clinical research
- Availability and details of other treatments and comparison with the anticipated benefits and

disadvantages of other treatments.

- Matters related to compensation and provision of medical care for damage to health caused by the implementation of specific clinical research
- (Matters to be reviewed by the Accredited Clinical Research Review Committee that conducts the business of reviewing and giving an opinion on the Specified Clinical Research and other matters concerning the Accredited Clinical Research Review Committee pertaining to said Specified Clinical Research)
- Other matters necessary for the implementation of specific clinical research

If, after the start of clinical research, the principal investigator obtains new findings related to the consent of research subjects and determines that changes to the explanation and consent documents are necessary, the principal investigator will revise them and submit them to an accredited clinical research review committee. The administrator of the institution will also be informed of the changes, together with the opinion of the Accredited Clinical Research Review Committee, and approval will be obtained.

New knowledge refers to new safety information or information on the development of new treatment methods, etc. for the disease.

## 13.4. informed consent

As the study involves pediatric patients, appropriate informed consent will be obtained from a legally authorized representative, and assent from the patient will be sought whenever feasible., the research subject and the surrogate consentor should be informed about the clinical research, given sufficient time to think about it, and asked to participate in the clinical research after confirming that the research subject and the surrogate consentor have a good understanding of the content of the clinical research. Consent by the surrogate will be as described in 13.5. When the research subject him/herself agrees to participate in the clinical research, a written consent document is used and signed by the research subject him/herself. The principal investigator or a subinvestigator confirms that the consent document includes the name of the physician who provided the explanation and the date of the explanation, the name of the research subject who received the explanation and consented, and the date of consent.

Two copies of the consent document should be made, one to be handed to the research subject and one to be kept by the principal investigator. The original should be kept in the medical record or in a storage place designated by the research institution.

Since personal information obtained in such clinical research is not specified at the time of obtaining consent and may be used for future research, consent to this effect should be obtained, and when conducting future clinical research, the research plan should be submitted to the Ethical Review Committee for review and reconfirmation of consent.

### 13.5. Consent by a Consenting Consenting Party

Since the disease subject to such clinical research is a research subject who is a minor, the following persons shall be selected as surrogate donors, based on the selection of persons who are considered to be able to represent the will and interests of the research subject, taking into consideration the family structure of the research subject, and the like.

Parents, siblings, children/grandchildren, grandparents, relatives living in the same household as the Research Subject, or persons considered to be equivalent to such close relatives (excluding minors) or the Research Subject's representative (including a voluntary guardian who has been granted the right to act on behalf of the Research Subject).

If consent is obtained from a surrogate, the relationship to the research subject should be noted on the consent form.

If the research subject is a minor 16 years of age or older and is able to fully understand the explanation, consent should be obtained from the research subject him/herself in addition to a surrogate. The items to be included in the consent form, storage, etc., shall be handled in accordance with 13.4.

### 13.6. informed assent

Since it is considered difficult for the patient himself/herself to give consent for the disease subject to the clinical research, the clinical research will be conducted with the consent of the surrogate. However, informed consent will be obtained when it is considered possible for the research subject to understand the explanations and express his or her willingness to participate in the clinical research with the assistance of a surrogate or other person. If the consent of the research subject differs from that of the surrogate, the surrogate's opinion shall be followed.

## 14. handling of personal information

All parties involved in the research shall comply with applicable laws, regulations, and ordinances regarding the protection of the personal information of research subjects. In addition, those involved shall make their utmost efforts to protect the personal information and privacy of research subjects, and shall not divulge any personal information obtained in the course of conducting this research without justifiable reason. The same shall apply even after the person concerned has retired from his/her position.

When handling samples, etc. related to the implementation of clinical research, sufficient consideration should be given to protecting the confidentiality of research subjects. When sending samples, etc. to the research secretariat or other related organizations, use a number, and give sufficient consideration so that the personal information of research subjects is not leaked outside the hospital. When publishing the results of clinical research, information that could identify the

research subjects should not be included. Samples, etc. of research subjects obtained in research will not be used for any purpose other than the purpose of clinical research.

A personal information manager shall be appointed at each practitioner institution. The Personal Information Manager shall be as shown in the Appendix.

# 15. deviations, changes or revisions to the research protocol

## 15.1. deviations or changes in the research protocol

The principal investigator or subinvestigator must not deviate from or change the research protocol before obtaining the approval of the hospital director based on the prior agreement of the principal investigator and the prior review by the Accredited Clinical Research Review Committee.

The principal investigator or subinvestigator may deviate from or change the research protocol for unavoidable reasons, such as emergency avoidance, before obtaining prior agreement with the principal investigator and prior approval from the accredited clinical research review committee. In such cases, the principal investigator or subinvestigator shall promptly submit to the principal investigator and the approved clinical research review committee the details of and reasons for the deviation or change and a draft of the revised research protocol, if necessary, and obtain the approval of the principal investigator, the approved clinical research review committee, and the hospital director.

The principal investigator or subinvestigator must record all deviations from the research protocol, along with the reasons for the deviations.

## 15.2. approval and revision of research protocol

The principal investigator submits the research plan to the administrator of the implementing medical institution prior to the start of the research, and obtains the approval of the accredited clinical research review committee and the administrator of the implementing medical institution for the implementation of the research.

When revising the research protocol, the principal investigator should consult with the study statistician and others as necessary regarding the appropriateness of the changes and their impact on the evaluation of the research before making a decision to revise the research protocol. In the event of revision, the revised research protocol must be submitted to the administrator of the site and approved by an accredited clinical research review committee and the administrator of the site.

After receiving approval for the revision, the principal investigator will promptly communicate the revised content to the parties involved in the study, including the research investigators and the data center.

# 16. termination and early termination of clinical studies

## 16.1. termination of clinical research

When the period for collecting data pertaining to all evaluation items described as matters related to the content of the clinical research has ended, the principal investigator shall prepare a summary report and its summary after modifying the implementation plan, in principle within one year from that date.

The summary report and its summary shall be submitted to the administrator of the implementing medical institution without delay after obtaining the opinions of the accredited clinical research review committee. The date of completion of the clinical trial shall be the date when the report is submitted to the Minister of Health, Labour and Welfare within one month from the date when the authorized clinical research review committee expressed its opinion, and the date when the report is made public by recording it in the jRCT.

## 16.2. early termination of clinical research

The clinical study will be terminated if any of the following are observed

- In view of the safety of the relevant clinical research and deviations from the authorities' regulations and the research protocol, a recommendation or instruction for discontinuation has been issued by an accredited clinical research review committee, or the principal investigator has determined that the research should be discontinued.
- Based on information other than the clinical research in question, a recommendation or instruction for discontinuation has been issued by an accredited clinical research review committee, or the principal investigator has determined that the study should be discontinued, because the safety of the study drug is considered to be problematic.
- Other reasons, such as delays in case enrollment and frequent deviations from the research protocol, made it difficult to complete the clinical research.
- When it is judged to be extremely difficult to achieve the planned caseload due to difficulties in recruiting research subjects.
- When the approved Clinical Research Review Committee has given instructions to change the implementation plan, etc., and it is deemed difficult to accept such instructions.

The principal investigator will promptly inform the research subjects and surrogates of the discontinuation and the reasons for it to ensure the safety of the research subjects.

The principal investigator will report in writing to the administrator of the medical institution, the accredited clinical research review committee, and the relevant department of the medical institution to which he/she belongs, and discontinue the study in accordance with the procedures established by the medical institution concerned.

Upon completion of the above, the principal investigator will notify the regulatory authorities of the discontinuation.

When clinical research is discontinued or suspended, the principal investigator must promptly

notify the research subjects and their surrogates to that effect, and take necessary measures, including the provision of appropriate medical care, after the discontinuation of clinical research.

# 17. report to the administrator of the medical institution

## 17.1. matters to be reported to the administrator of the executing medical institution

The principal investigator must report the following events to the administrator of his/her institution

- Noncompliance (reported to the site administrator by the research physician when there are concerns that are not reported to the site administrator by the principal investigator)
- Provision of research funds, etc. or other involvement by manufacturers and distributors of pharmaceuticals, etc.
- Opinion of the Accredited Clinical Research Review Committee
- Summary of Key Performance Indicators Report and Summary Report
- The publication of the summary of the main evaluation item report and the summary report.
- Submission of an implementation plan.
- Occurrence of serious adverse events that may be due to the conduct of the clinical research and subsequent
- defect
- Periodic Disease Reports
- periodic report

The principal investigator must obtain approval for the following events in addition to the report of the administrator of his or her institution.

- Whether or not to conduct clinical research
- Obtain consent from minors 16 years of age or older who meet certain criteria (no substitutes)

In the event that a research physician becomes aware of a nonconformity, he or she should promptly report it to the principal investigator.

## 17.2. matters to be reported to the Accredited Clinical Research Review Committee

The principal investigator must seek the opinion of or report the following events to an accredited clinical research review committee

- Start of research

- Occurrence of serious adverse events that may be due to the conduct of the clinical research and subsequent
- Periodic Disease Reports
- periodic report
- Change of implementation plan
- Changes to research protocols, conflict of interest management standards, and conflict of interest management plans
- Discontinuation of clinical research (if necessary)
- Critical Nonconformity
- Summary and publication of the Main Evaluation Item Report and Summary Report

### 17.3. matters to be reported to the Minister of Health, Labor and Welfare

The principal investigator must report the following events to the Minister of Health, Labour and Welfare (Director of the Regional Bureau of Health, Labour and Welfare).

- Implementation Plan (Study Initiation)
- Change of implementation plan
- Minor changes to the implementation plan
- Discontinuation of clinical research
- Completion of clinical research
- periodic report

The investigator must report the following events to the Minister of Health, Labour and Welfare (the President of the Pharmaceuticals and Medical Devices Agency).

- Disease reports (information on unanticipated serious adverse events that could be attributed to the conduct of clinical research in specific clinical research involving unapproved drugs and unapproved uses)

## 18. handling of materials related to clinical research

The principal investigator must submit all documents related to the implementation of clinical research, etc. (copies of application documents, notification documents from the approved clinical research review committee, notification documents from the administrator of the implementing medical institution, copies of various application forms and reports, research subject identification code list, (screening list), documents related to consent, documents related to registration, documents related to handling diseases, etc., copies of case reports, contracts related to the implementation of clinical research, records of acquisition and use, disposal, etc., of test drugs, test medical devices, test regenerative medical products, various procedures, monitoring reports, and other data. (e.g.,

documents pertaining to the implementation of the clinical research, documents pertaining to registration, documents pertaining to the response to diseases, etc., copies of case reports, etc., contracts pertaining to the implementation of the clinical research, records pertaining to the acquisition and use of test drugs, test medical devices, and test regenerative medical products and their disposal, various procedures, monitoring reports, and other documents or records necessary to ensure the reliability of the data). The electronic data and experimental/observational notes will be stored appropriately for a period of 10 years after the date of publication of the summary report in the jRCT or the date of final publication of the results of the relevant clinical research (e.g., in a paper), whichever is later, and other documents will be stored for a period of 5 years after that, after which they will be disposed of with care for personal information.

Medical records shall be stored and disposed of in accordance with the hospital's regulations.

- Research protocol, implementation plan, documents pertaining to the explanation to the subject of the specific clinical research and his/her consent, summary report, and other documents or copies thereof prepared by the principal investigator pursuant to the provisions of this Clinical Research Act and related ministerial ordinances.
- Documents received from accredited clinical research review committees pertaining to review opinion services
- Monitoring Documents
- Original documents, etc.
- Agreement for the Conduct of Specific Clinical Research
- Documents describing the outline of drugs, etc. to be used in the specified clinical research and records of acquisition and disposition of drugs, etc.

# 19. payment of money and compensation for conducting clinical research

## 19.1. expenses for clinical research

Ltd. will pay for the study drug Eppikajutsuto in this clinical research. Other than the above, the insurance portion of the study will be covered by the health insurance of the research subjects, so there will be no additional cost burden on the patients as a result of their participation in this study. Participation in the study is voluntary, and no honorarium will be paid to the research subjects. In addition, transportation to and from the hospital will be at the expense of the research subjects.

## 19.2. compensation for health hazards

Appropriate treatment will be provided in the event of any health hazard arising from the said clinical research. In the event of serious (to the extent that hospitalization is required) health problems arising from the proper use of the subject drug concerned, such problems will be covered by the "Indemnity Insurance for Clinical Research" to be taken out by the Research.

# 20. research funding and conflict of interest management

## 20.1. conflict of interest management plan

The Principal Investigator shall prepare a Conflict of Interest Management Standard and a Conflict of Interest Management Plan regarding possible conflicts of interest in this study, and obtain approval from the Accredited Clinical Research Review Committee. In addition, the principal investigator of this study will follow the "Enforcement Regulations of the Clinical Research Act" and the above management standards and management plan with regard to conflicts of interest. The principal investigator has a conflict of interest with Tsumura Corporation as an industry-academia collaborative activity, but the conflict of interest has been reviewed by the University's Conflict of Interest Management Committee and will not affect the conduct or results of this study. In conducting this study, data management was outsourced to a data center company in which the person in charge of clinical epidemiology (Hideki Ishikawa) is the sole shareholder.

## 20.2. sources of research funding

This research will be conducted with research and development funding provided by the Japan Agency for Medical Research and Development (AMED).

# 21. attribution of research results and publication of results

The summary of the primary endpoint report and the summary report shall be registered in jRCT and published within one month from the date of hearing the opinions of the accredited clinical research review committee. jRCT publication of the summary of the primary endpoint report and the summary report shall be conducted after the publication of the paper or other publication of the results of this research, after reporting to the accredited clinical research review committee that the paper or other publication is being submitted for publication. The publication by jRCT shall be made after the publication of the paper, etc., after reporting to the Accredited Clinical Research Review Committee that the paper, etc., is being submitted for publication.

The results of this research shall belong to the research group. Principal investigators and others will consult with each other to select authors and report the results at a conference or in a paper.

# 22. research organization

## 22.1. principal investigator

Professor Keiko Ogawa, Center for Chinese Medicine, Hiroshima University Hospital

Address: 1-2-3 Kasumi, Minami-ku, Hiroshima 734-8551

Phone: 082-257-5461

## 22.2. Principal Investigator

Keiko Ogawa, Professor, Center for Chinese Medicine, Hiroshima University Hospital
Address: 1-2-3 Kasumi, Minami-ku, Hiroshima 734-8551
Phone: 082-257-5461

Kiyosho Sakai, Project Assistant Professor, Department of Pediatric Surgery, Kanazawa University Hospital
13-1 Takara-machi, Kanazawa, Ishikawa 920-8641 Japan
Phone: 076-265-2947

Motonari Nomura, Osaka University Graduate School of Medicine
Address: 2-2 Yamadaoka, Suita-shi, Osaka 565-0871
Phone: (main) 06-6879-5111

Shuichiro Uehara, Chief, Department of Pediatric Surgery, Nihon University Itabashi Hospital
Address: 30-1, Oyaguchi Kami-cho, Itabashi-ku, Tokyo 173-8610, Japan
Phone: (main) 03-3972-8111

Naoki Shimojima, Director, Department of Pediatric Surgery, Division of Pediatric Surgery, National Center for Child Health and Development
Address: 2-10-1, Okura, Setagaya-ku, Tokyo 157-8535
Phone: (main) 03-3416-0181

St. Marianna University School of Medicine, Department of Surgery, Pediatric Surgery Kuma Ohbayashi
Address:〒 2-16-1 Sugo, Miyamae-ku, Kawasaki, Kanagawa 216-8511
Phone: (main) 044-977-8111

Showa University Hospital, Department of Surgery, Division of Pediatric Surgery Hideaki Sato
Address: 1-5-8 Hatanodai, Shinagawa-ku, Tokyo 142-8666
Phone: 03-3784-8789

## 22.3 Image Evaluation Committee to determine effectiveness

Keigo Osuga, Professor, Department of Diagnostic Radiology, Osaka Medical and Pharmaceutical

University Osaka Medical and Pharmaceutical University

Associate Professor Taiki Nozaki, Department of Radiology, School of Medicine, Keio University

## 22.4. Research Secretariat (Coordination and Management Practitioner)

Center for Chinese Medicine, Hiroshima University Hospital Eriko Kimura

〒1-2-3 Kasumi, Minami-ku, Hiroshima City 734-8551

TEL: 082-257-5461

FAX: 082-257-5461

mako1125@hiroshima-u.ac.jp

## 22.5. Data Management Officer

Atsuko Michishita, Manager, Medical Research Support Co.

Address: Koraibashi Yamamoto Building 6F, 3-1-14 Koraibashi, Chuo-ku, Osaka 541-0043, Japan

Phone: 06-6202-5446

FAX: 06-6202-5445

## 22.6. Trial Statisticians and Statistical Analysts

Yasushi Watahashi, Biostatistics Office, Hiroshima Clinical Research and Development Support Center, Hiroshima University Hospital

Address: 1-2 Kasumi, Higashihiroshima, Hiroshima 739-0046, Japan− 3

## 22.7. Clinical Epidemiology

Kyoto Prefectural University of Medicine Molecular Targeted Preventive Medicine

Specially Appointed Professor Hideki Ishikawa

465 Kaji-cho, Hirokoji-agaru, Kawaramachi-Dori, Kamigyo-ku, Kyoto, 602-8566, Japan

TEL: 075-251-5208

fax 075-211-7093

## 22.8. central monitoring officer

Medical Research Support Co.

Address: Koraibashi Yamamoto Building 6F, 3-1-14 Koraibashi, Chuo-ku, Osaka 541-0043, Japan

Phone: 06-6202-5446
FAX: 06-6202-5445

### 22.9. Case Registry Center

Medical Research Support Co.
6F Koraibashi Yamamoto Building, 3-1-14 Koraibashi, Chuo-ku, Osaka 541-0043, Japan
Phone: 06-6202-5446
FAX: 06-6202-5445

The registration details are checked against the selection and exclusion criteria in the research protocol, and the results are communicated to the principal investigator.

### 22.10. Person responsible for audit

Hiroshima University Hospital Hiroshima Clinical Research and Development Support Center Audit and Reliability Assurance Office Nobuyoshi Hiramatsu
Address: 1-2 Kasumi, Higashihiroshima, Hiroshima 739-0046, Japan− 3

## Complaints and Consultation

Keiko Ogawa, Center for Chinese Medicine, Hiroshima University Hospital
Address: 1-2-3 Kasumi, Minami-ku, Hiroshima 734-8551
Phone: 082-257-5461
FAX: 082-257-5461

## 24. literature

1) FY 2014-2018 Health and Labour Science Research Grants-in-Aid for Intractable Diseases (Intractable Disease Policy Research Project) "Research and Study on Intractable Hemangioma, Hemangiopericytoma, Lymphangioma, Lymphangiomatosis and Related Diseases" Group Hemangioma, Hemangiopericytoma, Lymphangiomatosis, Lymphangioma Medical Guidelines 2017 Second Edition March 31, 2017

2) Ogawa E, Higashimoto Y, Sekiya N, Terasawa S. A case of pediatric mediastinal lymphangioma successfully treated with Kampo therapy.

(3) Touko Shinkai, Koji Masumoto, Kazuki Shirane, Aya Ushiyama, Yasunari Tanaka, Tsubasa Aiyoshi, Masato Sasaki, Fumiko Chiba, Kentaro Ono, Hajime Kawakami, Shu Goto, Yasuhisa Urita, Hajime Takayasu Devices in Kampo Treatment of Lymphangioma in Combination with Koshuyu Kadzutsu The Japanese Journal of Pediatric Surgery 2019 55(1): 196-197

4) Hideaki Sato, Shigeyuki Furuta, Shutaro Manabe, Shiho Tsuji, Hiroaki Kitagawa Experience with

the use of Kesuyu Kadzhuyu for cystic lymphangioma (lymphatic malformation) Japanese Journal of Pediatric Surgery 2016 52(7):1290-1294

5) Hashizume N, Yagi M, Egami H, Asagiri K, Fukahori S, Ishii S, Saikusa N, Yoshida M, Masui D, Tanaka Y. Clinical Efficacy of Herbal Medicine for Pediatric Pediatr Dermatol. 2016 Mar-Apr;33(2):191-5. doi: 10.1111/pde.12777. Epub 2016 Jan 17.

(6) Nakahata Kengo, Ogawa-Ochiai Keiko, Yamanaka Hiroaki, Noguchi Yuki, Saka Ryuta, Takama Yuichi, Ueno Takehisa, Tazuke Yuko, Hidaka Kuniyuki, Osuga Keigo, Okuyama Hiroomi, A large cervical lymphatic malformation in a neonate successfully treated with Kampo medicine. Protocols: January 2021 2( 1 )0030
doi: 10.1097/MD9.0000000000000000030

7) Hiroaki Tanaka, Naoki Hashizume, Takahiro Asakawa, Tomomitsu Tsuru Three cases of adjuvant use of Eppikajutsuto for cystic lymphangioma Japanese Journal of Pediatric Surgery 2013 49(3): 821

8) Kunio Takano, Norio Hasuda, Fuminori Numano, Takeyuki Suzuki, Noboru Oyachi, Kozo Koshizuka Experience of Kampo treatment for pediatric cervical lymphangioma Japanese Journal of Pediatric Surgery 2015 51(2): 304-3059) Ogawa-Ochiai K, Sekiya N, Kasahara Y, Chino A, Ueda K Kimata Y, Yamamoto S, Okimoto Y, Higashimoto H, Namiki T, Terasawa K , A case of mediastinal lymphangioma successfully treated with Kampo medicine. J Altern Complement Med. 2011 Jun,17 (6):563-565. doi: 10.1089/acm.2010.0562. Epub 2011 May 13

(9) Nakahata, Kengo MD, PhDa; Ogawa-Ochiai, Keiko MD, PhDb,∗ ; Yamanaka, Hiroaki MD, PhDa; Noguchi, Yuki MD, PhDa; Saka, Ryuta MD, PhDa; Takama, Yuichi MD, PhDa PhDa; Ueno, Takehisa MD, PhDa; Tazuke, Yuko MD, PhDa; Hidaka, Kuniyuki MD, PhDc; Osuga, Keigo MD, PhDd; Okuyama, Hiroomi MD, PhDa A large cervical lymphatic malformation in a neonate successfully treated with Kampo medicine, Medicine Case Reports and Study Protocols: January 2021 - Volume 2 - Issue 1 - p e0030 doi: 10.1097/MD9.0000000000000000030

10) Haruna Makita, Kazuhira Endo, Akiko Shirai, Kanji Kawasaki, Rei Mishima, Yoshiya Kasahara, Kazuya Ishikawa, Takayoshi Ueno, Yosuke Nakanishi, Satoru Kondo, Naohiro Wakisaka, Tomokazu Yoshizaki, Keiko Ogawa-Ochiai, Eppikajutsuto (a Japanese herbal medicine) regulates mTOR and Satoru Kondo, Naohiro Wakisaka, Tomokazu Yoshizaki, Keiko Ogawa-Ochiai, Eppikajutsuto (a Japanese herbal medicine) regulates mTOR and induces apoptosis in oral Traditional & Kampo Medicine, 7(2):72-77(2020.8)
