# Supplementary material for: Eppikajutsuto for Treatment of Lymphatic Malformations in Children: A Nonrandomized Clinical Trial
Source: JAMA Netw Open. 2025 Nov 3;8(11):e2540897. doi: 10.1001/jamanetworkopen.2025.40897 (PMC12584033; doi:10.1001/jamanetworkopen.2025.40897)
Supplement: Supplement 2. — eTable. Composition of Eppikajyutsuto Extract Granules [file jamanetwopen-e2540897-s002.pdf]

Supplemental Online Content

Ogawa-Ochiai K, Sakai S, Saeki I, et al. Eppikajutsuto for treatment of lymphatic malformations in children: a nonrandomized clinical trial. *JAMA Netw Open*. 2025;8(11):e2540897. doi:10.1001/jamanetworkopen.2025.40897

eTable. Composition of Eppikajyutsuto Extract Granules

This supplemental material has been provided by the authors to give readers additional information about their work.

© 2025 Ogawa-Ochiai K et al. *JAMA Network Open*.

eTable. Composition of Eppikajyutsuto Extract Granules

Constituents	Weight (g)
Gypsum fibrosum	8
Ephedrae herba	6
Atractylodis lanceae	4
Zizyphi fructus	3
Glycyrrhizae radix	2
Zingiberis rhizoma	1

A 7.5 g portion of Tsumura eppikajyutsuto extract granules contains 5.0 g of dried extract of the following mixed crude drugs

© 2025 Ogawa-Ochiai K et al. *JAMA Network Open*.
