# Supplementary material for: Eppikajutsuto for Treatment of Lymphatic Malformations in Children: A Nonrandomized Clinical Trial
Source: JAMA Netw Open. 2025 Nov 3;8(11):e2540897. doi: 10.1001/jamanetworkopen.2025.40897 (PMC12584033; doi:10.1001/jamanetworkopen.2025.40897)
Supplement: Supplement 3. — Data Sharing Statement [file jamanetwopen-e2540897-s003.pdf]

# Data Sharing Statement

Ogawa-Ochiai. Eppikajutsuto for Treatment of Lymphatic Malformations in Children. *JAMA Netw Open*. Published November 03, 2025. doi:10.1001/jamanetworkopen.2025.40897

**Data**
**Additional Information:** Japan Registry of Clinical Trials (jRCTs041210007, 2021/04/16) https://jrct.mhlw.go.jp/en-latest-detail/jRCTs041210007
**Data available:** No
